# InSAR Techniques for Landslide Study: A Review of Methods, Challenges, and Emerging Trends

**DOI:** 10.3390/s26154667

**Published:** 2026-07-23

**Authors:** Hanlu Zhang, Bowen Liu, Daming Zhu, Zhanfeng Liu, Mengyao Shi

**Affiliations:** 1 Faculty of Land Resources Engineering, Kunming University of Science and Technology, Kunming 650031, China; hanlu_zhang@stu.kust.edu.cn (H.Z.); shimengyao@kust.edu.cn (M.S.); 2 Yunnan Key Laboratory of Intelligent Monitoring and Spatiotemporal Big Data Governance of Natural Resources, Kunming 650051, China; 18087752215@126.com; 3 Key Laboratory of High Impact Weather (Special), Changsha 410073, China; 15808712152@163.com; 4 Yunnan Meteorological Observatory, Kunming 650034, China; 5 Yunnan Institute of Geology and Mineral Surveying and Mapping Co., Ltd., Kunming 650051, China

**Keywords:** landslide monitoring, InSAR, deep learning, surface deformation, rheology

## Abstract

Landslides occur frequently under complex and variable global geomorphological and meteorological conditions, posing serious threats to the ecological environment, human life and property. Traditional monitoring approaches are often inefficient and highly susceptible to external conditions, making them inadequate for large-scale rapid deformation monitoring. Owing to its advantages of high precision, wide-area coverage, and all-weather continuous observation, Interferometric Synthetic Aperture Radar (InSAR) technology has been widely applied in landslide monitoring. This paper systematically reviews the development and application of InSAR technologies in landslide monitoring. Classical methods, including Differential InSAR (D-InSAR), Permanent Scatterer InSAR (PS-InSAR), Small Baseline Subset InSAR (SBAS-InSAR), and Distributed Scatterer InSAR (DS-InSAR), as well as derivative techniques such as Quasi-Permanent Scatterer InSAR (QPS-InSAR), Temporarily Coherent Point InSAR (TCP-InSAR), and Multiple Aperture InSAR (MAI), are comprehensively summarized. In addition, recent advances in artificial intelligence (AI) and multi-source data fusion are highlighted. Through comparative analysis of related studies, this paper summarizes the applicability and potential of various methods in landslide monitoring, and reviews the main solutions to challenges, including geometric distortion, decorrelation noise, atmospheric delay, and difficulties in three-dimensional deformation monitoring. Future research directions are also discussed. Overall, InSAR technology has evolved from single-method approaches toward integrated and intelligent frameworks; however, challenges remain in terms of adaptability in complex terrain, data-processing efficiency, and model interpretability. This review provides a technical reference for future landslide-monitoring research.

## 1. Introduction

Geological hazards are among the primary natural threats to ecological security and human activities in mountainous regions. Among them, landslides occur frequently and are triggered by complex factors, making them one of the most widely distributed geological hazards worldwide [1]. From a scientific perspective, a landslide refers to the downslope movement of rock, debris, or soil under the influence of gravity, whereas a landslide disaster denotes an event in which such movement causes damage to exposed elements, including human populations, infrastructure, and ecosystems [2,3]. Not all landslides evolve into disasters; their destructive potential depends on factors such as the scale of the landslide, its kinematic characteristics, its spatial location, and the vulnerability of the exposed elements [4]. This distinction defines the core objective of landslide monitoring: not only to identify deformation processes within landslide bodies, but also to prevent their evolution into disasters through early detection and risk assessment. Landslides result from the combined influence of multiple factors, including natural drivers such as geological structures, geomorphology, earthquakes, and climatic conditions, as well as anthropogenic activities such as mining, hydraulic engineering, and vegetation destruction [5]. Landslides are characterized by wide spatial coverage, strong concealment, and high destructive potential. Once they develop into disasters, they can pose serious threats to regional ecological environments, public infrastructure, and the safety of human lives and property [6]. Therefore, strengthening landslide monitoring and early warning is of critical importance.

Traditional landslide deformation-monitoring methods, such as inclinometer monitoring [7], optical remote sensing monitoring [8], and oblique photogrammetry monitoring [9], can provide important data support to a certain extent, but they also exhibit several notable limitations. These methods generally require long monitoring periods and have limited spatial coverage, resulting in low efficiency in large-scale monitoring applications. In addition, they are easily constrained by practical conditions such as weather and terrain, which can affect the accuracy and timeliness of the acquired data [10]. Consequently, traditional monitoring approaches struggle to meet the demands for rapid and comprehensive surface deformation-monitoring [11]. To improve the accuracy of landslide monitoring and early-warning capabilities, extensive research has been conducted by scholars worldwide on landslide hazard prevention, monitoring, and the development of risk management frameworks, leading to substantial practical experience and technical expertise. Among these approaches, InSAR, as an emerging remote sensing monitoring technology [12,13], has been widely applied and extensively studied in landslide hazard monitoring due to its advantages, including high monitoring accuracy, wide spatial coverage, non-contact measurement, relatively low overall cost, and the ability to conduct continuous observations regardless of weather and time conditions [14,15,16]. InSAR technology is highly suitable for the early identification of landslide hazards. By detecting subtle surface deformations, it enables continuous monitoring of landslides over large areas, revealing the spatial characteristics and evolutionary processes of landslide deformation [17]. In addition, InSAR can provide periodically updated surface displacement information, offering important data support for landslide risk assessment and disaster early warning.

Researchers worldwide have advanced the application of InSAR in landslide monitoring from multiple technical perspectives. In terms of multidimensional deformation monitoring, two major technical approaches have been developed. The first approach integrates multi-orbit InSAR with Pixel Offset Tracking (POT) or MAI, thereby introducing azimuthal deformation observations to construct three-dimensional inversion equations [18]. For example, Ref. [19] incorporated POT and related techniques to reconstruct the three-dimensional displacement field of the Hooskanaden landslide. The second approach introduces prior constraints, such as neglecting north–south deformation components [20] or adopting the surface-parallel flow assumption [21]. However, mountainous landslides are often affected by layover and shadow effects, resulting in the availability of only single-orbit observations and consequently limiting the retrieval of three-dimensional deformation information. In terms of multi-band data fusion and inversion, Synthetic Aperture Radar (SAR) datasets from different frequency bands, such as L-, C-, and X-band, can be jointly utilized to exploit the complementary advantages of long wavelengths with strong vegetation penetration and short wavelengths with high revisit frequency. This strategy helps mitigate decorrelation in vegetated areas while improving spatiotemporal monitoring coverage and inversion accuracy [22]. Studies [23,24] demonstrated the coherence advantages of L-band SAR under vegetated conditions. Reference [25] further quantitatively analyzed the temporal decorrelation characteristics of surface-covered areas using L-, C-, and X-band SAR data, clarifying the coherence attenuation patterns of different frequency bands. Reference [26] established a quantitative decorrelation estimation model based on Landsat-derived NDVI, enabling the prediction of monitoring performance in vegetated areas prior to SAR data acquisition. These studies provide theoretical and methodological support for multi-band fusion inversion and optimal band selection. Although multi-band data fusion has achieved certain applications in landslide monitoring, the underlying patterns and physical mechanisms embedded in the data remain insufficiently explored, leaving substantial room for future improvement. Regarding multi-factor coupling modeling and mechanism interpretation, significant progress has been made in coupling analyses between InSAR-derived deformation and triggering factors such as rainfall, earthquakes, and reservoir water levels [27,28,29]. Representative examples include the lagged response relationship between rainfall peaks and landslide displacement [30], as well as landslide deformation induced by human activities [31]. These studies have jointly promoted the coupling modeling of InSAR deformation and external triggering factors, providing important support for landslide mechanism interpretation and early warning. Nevertheless, the application of InSAR technology in landslide hazard monitoring still faces many challenges. Owing to terrain and environmental constraints, issues such as decorrelation noise, atmospheric disturbances, and the inability to comprehensively retrieve three-dimensional deformation information frequently occur [32].

In recent years, review studies on InSAR-based landslide monitoring have made significant progress. Bhattacharya et al. reviewed the application of InSAR technology in landslide monitoring in the Indian Himalayas, systematically summarizing the technical challenges and potential solutions under complex terrain conditions [33]. Coutinho et al. assessed the applicability of InSAR technology for slope monitoring, analyzing the feasibility and limitations of the method from an engineering perspective [34]. Cheng et al. focused on the advancements of spaceborne InSAR in landslide monitoring and susceptibility mapping, providing a systematic overview of technological developments such as atmospheric correction and three-dimensional deformation monitoring [35]. These review studies have advanced the field from different perspectives, including regional applications, engineering practice, and technological development. However, the evolutionary trajectory of InSAR techniques themselves still requires further systematic clarification, while more targeted discussions on key scenarios such as landslide hazard prevention and mitigation remain necessary. In addition, the integrated analysis of classical methods and emerging technologies still needs to be strengthened.

Based on this background, this review focuses on the application of InSAR techniques in landslide monitoring, with the evolution of methodologies as the central theme. This review is structured around three aspects: technological evolution, expansion of application scenarios, and emerging research directions. First, the development of classical InSAR approaches (D-InSAR, PS-InSAR, SBAS-InSAR, and DS-InSAR) and their derivative techniques is systematically reviewed. Second, combined with representative case studies, the typical application scenarios of InSAR technology in landslide deformation monitoring and disaster prevention are summarized. Third, recent advances in multi-source data fusion and the integration of artificial intelligence with InSAR are highlighted. On this basis, key technical challenges—including geometric distortion, decorrelation, atmospheric delay, and three-dimensional deformation monitoring—are analyzed. By comparing with existing reviews, this study clarifies its focus on systematically synthesizing the technological evolution of InSAR and integrating emerging research directions, with the aim of providing a useful reference for future studies. Considering that spaceborne InSAR is currently the most widely used approach in landslide-monitoring research and applications, this review mainly focuses on the application of spaceborne (satellite-based) InSAR in landslide monitoring.

## 2. Data Sources and Processing for Landslide Monitoring

### 2.1. Data Sources

SAR data constitute the core information source for achieving precise landslide monitoring using InSAR technology. Sensor characteristics, including sensor type, spatial resolution, and revisit cycle, directly affect the accuracy and timeliness of deformation monitoring [36]. At present, various SAR satellites can provide multi-band and multi-polarization observation data to meet the monitoring requirements of different terrain conditions and landslide types. According to their operational characteristics and mission objectives, SAR satellites can generally be classified into military, civilian, and commercial categories. Among them, military satellite data are usually inaccessible to the public, civilian satellites mainly adopt open-access data-sharing policies, whereas commercial satellites provide customized observation services with high spatial resolution and high revisit frequency. Table 1 and Table 2 summarize the key parameters and application scenarios of several commonly used operational SAR satellites. Sentinel-1A, ALOS-2, and Radarsat-2 are selected as representative examples to analyze how differences in radar wavelength, revisit interval, and spatial resolution influence the suitability of SAR data for different landslide-monitoring scenarios.

Sentinel-1A is one of the most widely used civilian SAR satellites with an open data policy and serves as a representative platform for C-band SAR observations [37]. Equipped with a C-band SAR sensor, it supports dual-polarization (VV + VH) imaging. Its short revisit interval enables timely detection of dynamic landslide deformation, making it particularly suitable for monitoring rapidly evolving landslides triggered by heavy rainfall, earthquakes, and other extreme events [38]. In addition, the dual-polarization mode distinguishes the surface scattering characteristics of landslides from those of surrounding stable areas by analyzing the differences between polarization channels, thereby providing complementary information for deformation signal extraction [39]. The study in Reference [40] demonstrated that the high-coherence data and frequent observation capability of the Sentinel-1 satellites enable effective detection of potential landslide deformation during periods of intense rainfall. This strongly corresponds to the role of heavy rainfall as a major triggering factor for landslides and highlights the significant advantages of Sentinel-1 data for the early identification of regional-scale landslide hazards.

ALOS-2 is a representative L-band SAR mission and serves as an important data source for landslide monitoring in vegetated regions [41]. Compared with C-band systems, its longer wavelength provides stronger vegetation penetration capability, effectively reducing the attenuation of radar signals caused by dense vegetation and enabling the retrieval of actual ground deformation information beneath vegetation cover [42]. This characteristic gives ALOS-2 irreplaceable advantages in landslide monitoring over mountainous and forested regions with complex terrain, addressing the severe coherence loss commonly encountered by C-band satellites in densely vegetated areas. Reference [43] pointed out that the Phased Array type L-band Synthetic Aperture Radar (PALSAR) instrument onboard the ALOS satellite demonstrates high applicability for InSAR-based landslide monitoring due to its high spatial resolution, multi-mode observation capability, systematic acquisition strategy, and rigorously calibrated and validated data quality. These features provide continuous and reliable data support for long-term landslide deformation monitoring.

Radarsat-2 is a representative commercial SAR satellite that supports multiple imaging modes with different spatial resolutions, making it an important platform for small-scale landslide monitoring and detailed deformation analysis [44]. The satellite supports four polarization modes (HH, HV, VH, and VV) and multiple resolution configurations, allowing for flexible adjustment of acquisition parameters according to landslide scale and monitoring accuracy requirements. For small landslides or localized deformation within a landslide body, high-resolution modes can be selected to accurately delineate landslide boundaries and capture subtle deformation features [45]. For regional-scale landslide screening, parameter settings can be optimized to balance coverage and efficiency [46]. The study in Reference [47] demonstrated that high-resolution InSAR based on Radarsat-2 enables precise measurement of landslide displacement, with results showing strong consistency with Global Positioning System (GPS) observations, and displacement variations in stable areas within ±1 mm. The study successfully identified six active landslide zones using satellite data, with average displacement rates ranging from 11 to 39 mm/year, and revealed the seasonal variability of landslide deformation. These findings provide key evidence for landslide hazard risk classification and management decision-making.

Overall, wavelength, revisit interval, and spatial resolution jointly determine the suitability of SAR data for different landslide-monitoring scenarios. Regarding wavelength, it influences the selection of monitoring targets by affecting vegetation penetration capability and interferometric coherence. L-band SAR is more suitable for landslide monitoring in densely vegetated mountainous regions, whereas C-band SAR is generally more effective for large-scale deformation monitoring in bare or sparsely vegetated areas [48]. X-band SAR systems (e.g., TerraSAR-X), benefiting from their higher spatial resolution, are more appropriate for detailed deformation monitoring in urban areas and regions surrounding critical infrastructure [49]. In terms of revisit interval, longer revisit periods are suitable for analyzing long-term deformation trends of slow-moving landslides, while shorter revisit intervals are advantageous for capturing rapid deformation processes associated with triggering factors such as heavy rainfall and earthquakes. Regarding spatial resolution, medium- and low-resolution SAR data are suitable for regional-scale landslide hazard screening, whereas high-resolution SAR data are better suited for individual landslide boundary delineation and subtle deformation monitoring. In practical applications, SAR data should be selected by considering landslide characteristics, topographic and vegetation conditions, and monitoring objectives. When necessary, multi-source SAR observations can be integrated to achieve a balance among spatial coverage, temporal continuity, and deformation-monitoring accuracy.

In recent years, high-revisit commercial SAR constellations represented by ICEYE and Capella Space have developed rapidly. Through multi-satellite networking, these systems have significantly shortened revisit intervals, providing new operational pathways for near-real-time disaster monitoring [50]. ICEYE data have been applied to the rapid monitoring of natural disasters such as floods and earthquakes [51], while Capella Space has deployed the first operational commercial SAR satellite, enabling flexible task scheduling and high-resolution imaging capabilities [52]. These satellite systems offer advantages such as rapid task responsiveness, high observation flexibility, and frequent data updates, making them particularly suitable for post-earthquake landslide detection, emergency monitoring of rainfall-induced landslides, and continuous deformation tracking. However, due to limitations in data cost and spatial coverage, current commercial constellations are mainly used for emergency response and disaster relief missions, and have not yet achieved routine operational applications at regional scales. In the future, with the continued expansion of domestic and international commercial SAR constellations, they are expected to complement traditional civilian satellites and gradually integrate into operational landslide monitoring and early-warning systems.

### 2.2. Data Acquisition and FAIR Principles

From the perspective of data characteristics, continuous observations from multi-source SAR satellites have generated TB- to PB-scale datasets. Raw echo data, single-look complex (SLC) images, and derived interferometric products exhibit typical big data characteristics, including large data volume (Volume), rapid data growth (Velocity), and high data dimensionality (Variety), which pose significant challenges for efficient storage, management, and analysis [53].

Regarding data acquisition, civilian SAR data (e.g., Sentinel-1, ALOS-2, and NISAR) are mainly provided free of charge through official portals of space agencies, such as the ESA Copernicus Open Access Hub, JAXA G-Portal, and ASF DAAC, and are generally available through a registration-based access mechanism. In contrast, commercial SAR data (e.g., Radarsat-2, ICEYE, and Capella Space) typically require purchase from satellite operators or authorized distributors, although some providers offer sample datasets or scientific collaboration programs. Raw SAR products are commonly distributed in the form of Level-0 (raw echo data) or Level-1 (SLC) products, and users need to select appropriate product levels according to specific monitoring requirements.

The FAIR data principles (Findable, Accessible, Interoperable, and Reusable) provide a fundamental framework for scientific data management and sharing [54]. Findability requires standardized metadata descriptions and unique identifiers to facilitate the retrieval of SAR data for specific regions and time periods. Accessibility emphasizes open data policies that reduce barriers to scientific research and enable institutions and individuals with limited resources to access data resources. Interoperability requires consistent data formats, coordinate reference systems, and processing standards to support the integration and joint analysis of multi-source SAR datasets. Reusability requires comprehensive processing histories and quality information to enable independent validation and further scientific investigations [55,56]. Open SAR data-sharing policies (e.g., Sentinel-1 and NISAR) can reduce the cost of repeated data acquisition, promote international collaboration in global landslide-monitoring research, and enhance both scientific reproducibility and economic efficiency.

### 2.3. Data Preprocessing Procedures

**Figure 1:**
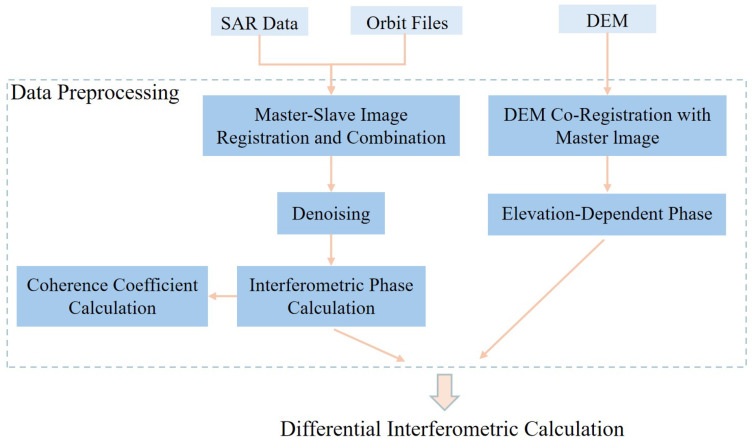
InSAR Data Preprocessing Workflow.

In a broad sense, data preprocessing includes image registration and coregistration, Digital Elevation Mode (DEM) alignment, noise reduction, interferometric phase computation, flat-earth and topographic phase removal, filtering, and phase unwrapping. Since different InSAR techniques exhibit significant differences in the differential interferometry processing stage, this paper defines data preprocessing as the common steps prior to interferogram generation to maintain the integrity and coherence of subsequent methodological descriptions. The differential interferometry computation and subsequent processing are presented in the corresponding methodological sections. The overall workflow is shown in Figure 1. First, master image selection, image registration, and coregistration are performed. Then, the DEM is aligned with the master image to generate the elevation-dependent phase, which is used for subsequent flat-earth and topographic phase removal. Next, noise reduction is applied to mitigate the impact of speckle noise on interferometric measurements. After that, interferometric phase and coherence coefficient are computed to generate the initial interferogram and coherence map. Finally, the process enters the differential interferometry stage (Section 3.1, Section 3.2, Section 3.3 and Section 3.4), where surface deformation information is further extracted. In the following, several key data preprocessing steps are briefly described in the context of landslide-monitoring applications.

(1) Master image selection, coregistration, cropping, and stacking: By calculating the temporal and spatial baselines for all image pairs, temporal and spatial baseline distribution maps are generated. Master image selection should be differentiated according to landslide types [57,58]. In areas with dense vegetation, images from the dry season with stable atmospheric conditions are prioritized as master images to reduce atmospheric delay interference on deformation signals; in rapidly deforming landslide zones, images near the mid-deformation period are preferred to balance coherence and deformation detectability [59]. Based on this, all SAR images are coregistered and cropped to the master image, and stacked to generate a time-series interferogram collection. In landslide regions, the coherence maximization method is typically used to optimize coregistration accuracy, and control point density is increased in high-deformation areas to ensure precise coregistration [60].

(2) DEM-to-master image registration: DEM-to-master image registration involves transforming an external DEM into the SAR image coordinate system. By simulating SAR intensity images from the DEM and coregistering them with the actual master image, a geometric mapping between the two can be established. The registration accuracy directly affects the precision of topographic phase removal, which in turn determines the reliability of differential interferometry. The InSAR-DEM joint adjustment method proposed by Hong et al. can reduce elevation errors by 50–95% and improve horizontal accuracy by 83–93% [61]. For complex terrains similar to the Qinghai-Tibet Plateau in landslide monitoring, high-resolution DEMs (e.g., Light Detection and Ranging (LiDAR) DEM, Copernicus 30 m DEM) are recommended to minimize topographic residuals [62], and external control points can be introduced if necessary to further enhance registration accuracy.

(3) Denoising: SAR images are affected by noise during acquisition and transmission, which impacts interferometric measurement accuracy and thus requires denoising. Common methods include multilooking and filtering techniques [63]. Multilooking reduces speckle noise by averaging neighboring pixels but decreases spatial resolution; when processing landslide data, the multilook factor should be chosen according to landslide size and resolution requirements. Filtering techniques can be applied in the spatial domain (e.g., mean or median filtering) or frequency domain (e.g., wavelet transform filtering), and the appropriate method should be selected based on the type and distribution of noise. For example, uniformly distributed speckle noise is suitable for median filtering, whereas frequency-domain noise with specific characteristics may be addressed using wavelet transform filtering [64]. In [65], the Non-Local InSAR (NL-InSAR) adaptive filtering technique was proposed and applied, improving scatterer detection accuracy in vegetated or complex terrains and significantly enhancing the quality of interferometric measurements of persistent scatterers.

(4) Interferometric phase computation: Interferometric phase computation is a core step in InSAR data preprocessing, which derives surface deformation information by calculating the phase difference between coregistered master and slave images. In practice, the coregistered master and slave images are first prefiltered in the frequency domain by extracting the common bandwidth, and then each pixel of the filtered images is multiplied by the complex conjugate of the corresponding pixel to generate the interferogram [66]. Landslide-monitoring areas are often affected by widespread decorrelation and phase discontinuities. Therefore, spatial filtering and adaptive filtering methods are commonly adopted to improve interferogram quality. During the filtering process, the filter window size and strategy should be adjusted according to coherence conditions [67]. Studies indicate that filtering windows ranging from 48 × 48 to 72 × 72 pixels achieve an optimal balance between noise suppression and preservation of deformation information, effectively retaining deformation features and aiding accurate identification of landslide hazards. For high-precision monitoring of small-scale landslides, the window size must be strictly controlled to avoid boundary blurring [68].

## 3. InSAR Methods for Landslide Monitoring

**Figure 2:**
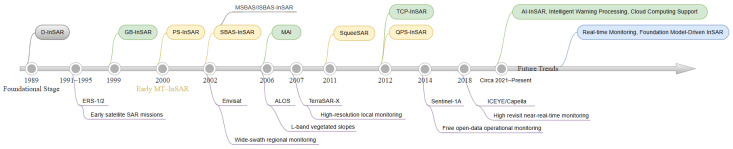
Development History of InSAR Techniques for Landslide Monitoring. Colors indicate development stages: gray for conventional D-InSAR; yellow for early MT-InSAR; green for expansion, hybrid and auxiliary integrated methods; and blue for future trends.

Since the 1990s, InSAR technology has played a central role in landslide hazard monitoring. Its development has evolved from conventional D-InSAR to Multi-Temporal InSAR (MT-InSAR), followed by the continuous advancement toward intelligent and near-real-time monitoring. Throughout this evolution, key challenges such as spatial–temporal decorrelation and atmospheric delay have been progressively mitigated, leading to significant improvements in monitoring accuracy and reliability [69]. Figure 2 summarizes the development trajectory of InSAR technologies in landslide monitoring, from early-stage methods to intelligent applications.

Conventional D-InSAR laid the foundation for remote sensing–based landslide deformation monitoring; however, it is limited by coherence loss and atmospheric effects and cannot effectively retrieve time-series deformation information [70]. To address these limitations, MT-InSAR techniques were developed along two major directions. The first is PS-InSAR [71], which achieves high-precision long-term monitoring based on stable point targets. The second is DS-InSAR, which focuses on distributed scatterers and has led to the development of methods such as SBAS-InSAR [72] and SqueeSAR, thereby improving the point density and reliability of nonlinear deformation monitoring in natural areas [73]. Meanwhile, technologies such as Ground-Based InSAR (GB-InSAR) and MAI have further expanded the capability for local high-frequency and three-dimensional deformation monitoring. With continued research progress, MT-InSAR has further entered an integrated development stage. TCP-InSAR [74] and hybrid PS + SBAS/DS approaches, such as Stanford Method for Persistent Scatterers (StaMPS) and Adaptive Distributed Scatterer InSAR Combined with Land Cover (ADSI-CLC), have gradually emerged, and integrated inversion frameworks combining multiple methods have become an important research focus [75]. With the continuous advancement of SAR satellite platforms, missions such as ERS, Envisat, ALOS, and TerraSAR-X have progressively expanded the spatial coverage, application scenarios, and refinement level of landslide monitoring [76,77]. Following the launch of Sentinel-1 in 2014, its open-access data policy and stable revisit capability significantly improved the operationalization and continuity of regional-scale landslide monitoring [78]. In recent years, InSAR technology has further evolved toward intelligent, automated, and high-frequency observation systems. Multi-sensor fusion approaches integrate SAR data from different frequency bands and platforms while incorporating auxiliary information from optical remote sensing and Global Navigation Satellite System (GNSS) to establish space–air–ground integrated monitoring networks [79]. In addition, machine learning and deep learning techniques, such as Convolutional Neural Networks (CNNs) and Long Short-Term Memory networks (LSTMs), have substantially improved the automation and accuracy of phase unwrapping, atmospheric correction, and deformation prediction [80]. At the same time, commercial SAR constellations such as ICEYE and Capella have greatly shortened revisit intervals, enabling high-frequency and near-real-time surface deformation monitoring and providing strong support for the timely detection and early warning of landslide hazards [81].

In summary, InSAR technology for landslide hazard monitoring has evolved from early D-InSAR into a comprehensive technical framework encompassing PS-InSAR, SBAS/DS-InSAR, TCP-InSAR, GB-InSAR, MAI, and intelligent processing approaches [73,74,75]. These technologies are capable of addressing landslide deformation-monitoring challenges under diverse environmental conditions. In the future, with the deeper integration of artificial intelligence, the development of high-revisit satellite constellations, and advances in multi-platform collaborative observation, InSAR is expected to continue evolving toward greater accuracy, intelligence, and operational applicability.

These methods each have their own advantages in terms of monitoring accuracy, applicable scenarios, and technical characteristics, and a thorough understanding of their properties is essential for optimizing landslide-monitoring strategies. In the following sections, this paper will provide a detailed explanation of the core methods and briefly introduce the characteristics and applicability of representative derivative techniques.

### 3.1. D-InSAR Method

**Figure 3:**
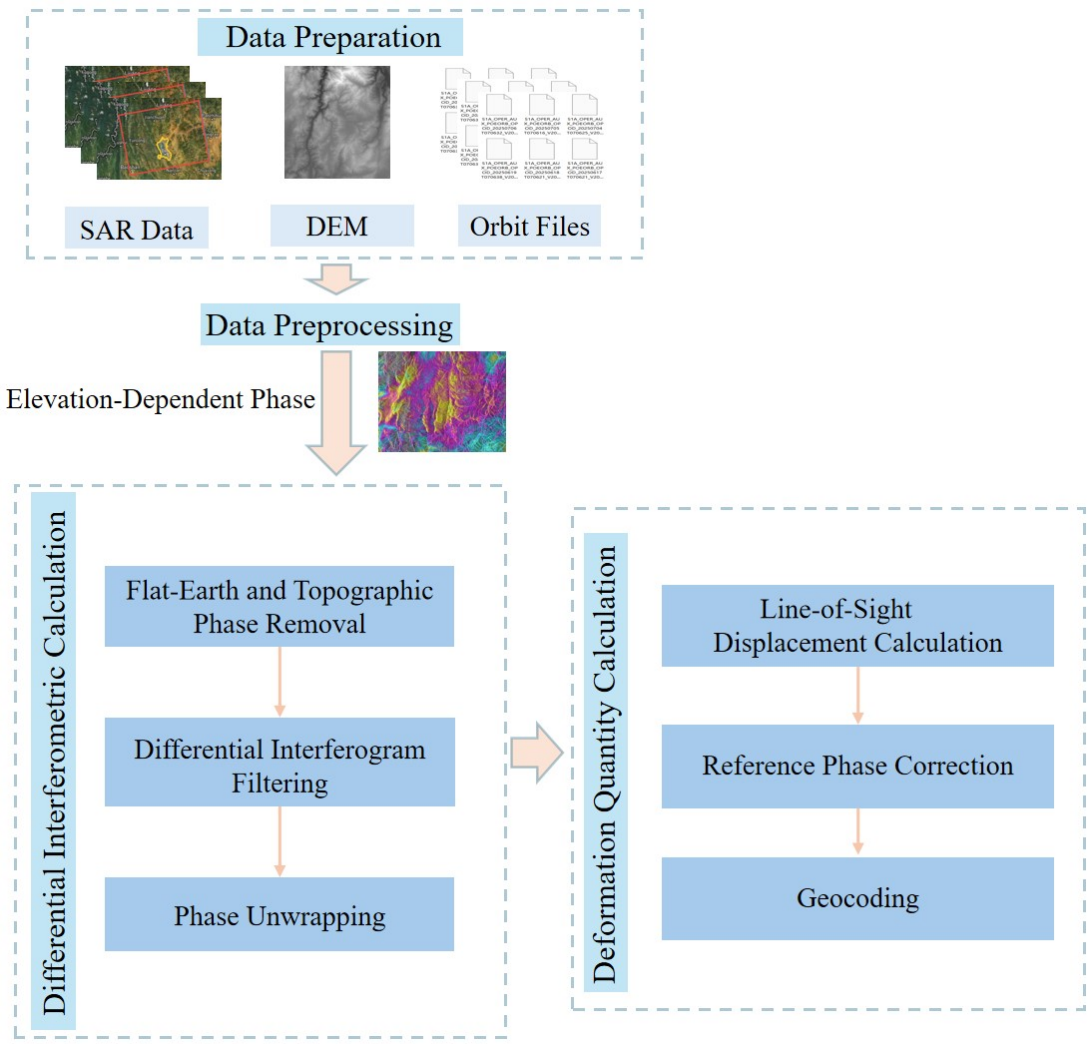
D-InSAR Processing Workflow.

D-InSAR is a remote sensing technique developed based on conventional InSAR methods. This technique interferes SAR data acquired over the same area at different times, and by applying differential processing to remove common phase components between the two observations, it retrieves information on surface deformation [82]. The specific workflow is illustrated in Figure 3: first, the acquired SAR data are preprocessed (see Section 2.2 for details on data preprocessing); then, the computed elevation phase is used to remove both flat-earth and topographic phases, generating differential interferograms that are filtered to reduce noise and other interferences; Next, phase unwrapping is performed to recover the true continuous phase values. Subsequently, reference correction is applied by selecting control points to refine orbital parameters; Finally, the processed data are geocoded to produce deformation maps.

D-InSAR requires only two SAR images to complete interferometric processing, making it particularly suitable for short-term rapid landslide deformation monitoring triggered by sudden factors such as heavy rainfall and earthquakes. Its processing workflow is relatively simple, enabling rapid disaster response and large-scale monitoring [83]. Reference [71] applied D-InSAR in the Alpine region, characterized by rugged terrain and high surface coherence, to monitor rockslide scenarios, effectively detecting subtle instabilities (with centimeter-level accuracy) that are difficult to identify with conventional ground-based methods, demonstrating its advantage for large-scale continuous coverage. However, D-InSAR has limitations: it struggles to measure high spatial gradient movements, exhibits low coherence in densely vegetated areas, is unsuitable for long-term analysis, assumes linear displacement, cannot capture three-dimensional motion, and is sensitive to geometric distortions and noise [84]. Therefore, multi-temporal InSAR techniques were developed to overcome these limitations.

### 3.2. PS-InSAR Method

**Figure 4:**
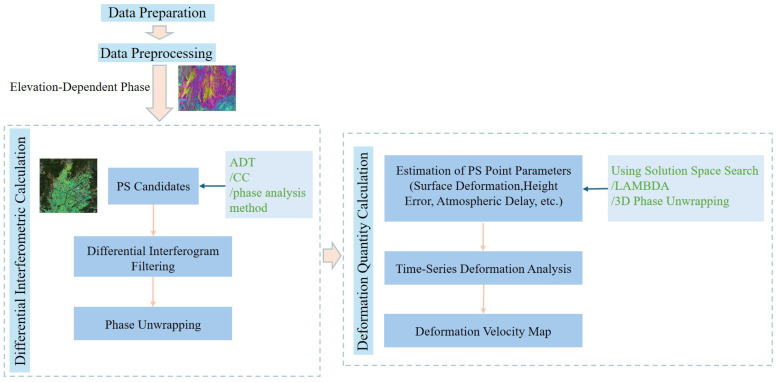
PS-InSAR Processing Workflow.

PS-InSAR utilizes ground targets with stable backscattering characteristics over long periods, such as buildings and rocks, as Permanent Scatterers (PS). By processing and analyzing multi-temporal SAR datasets acquired over the same area, surface deformation information can be retrieved. The general workflow is illustrated in Figure 4: unlike D-InSAR, stable PS points must first be identified from interferograms using methods such as the Amplitude Dispersion Threshold (ADT), coherence coefficient (CC), and phase analysis approaches [69,85]. After generating the differential interferograms, parameters including landslide deformation and elevation error are estimated for the selected PS points. Commonly used approaches include solution space search methods [69], the Least-squares AMBiguity Decorrelation Adjustment (LAMBDA) method [86], and the three-dimensional unwrapping method implemented in StaMPS [87]. Subsequently, time-series deformation analysis is conducted to estimate long-term landslide deformation rates.

PS-InSAR is better suited for long-term time-series data with relatively large spatial and temporal baselines, effectively alleviating the susceptibility of conventional D-InSAR to spatial–temporal decorrelation and atmospheric disturbances, while significantly improving deformation-monitoring accuracy [88]. Consequently, it has been widely applied to the early identification of landslides exhibiting long-term slow deformation. For example, Reference [89] employed PS-InSAR with Sentinel-1 data to achieve near-real-time, automated, and long-term monitoring of landslides in the Tuscany region of Italy, successfully identifying deformation acceleration induced by seasonal rainfall. This study demonstrated the advantages of PS-InSAR for regional-scale monitoring of slow-creeping landslides, with monitoring accuracy reaching the millimeter to sub-millimeter level [90]. However, PS density is strongly influenced by surface cover conditions. In urban areas, PS density can reach 100–300 PS/km^2^ (e.g., an average of 150 PS/km^2^ in the Los Angeles Basin), whereas it decreases significantly in vegetated regions (e.g., only about 41 PS/km^2^ in the Ancona landslide area) [91,92]. Insufficient PS density directly reduces the spatial resolution and reliability of deformation inversion; therefore, PS-InSAR is more suitable for urban and hard-target areas [93]. In addition, the technique also suffers from limitations such as low computational efficiency and limited applicability for large-scale monitoring [94]. To address these issues, reference [95] proposed the StaMPS, which improves the PS extraction strategy and significantly enhances the applicability of PS-InSAR in complex surface environments, thereby laying the foundation for landslide monitoring in mountainous areas.

### 3.3. SBAS-InSAR Method

**Figure 5:**
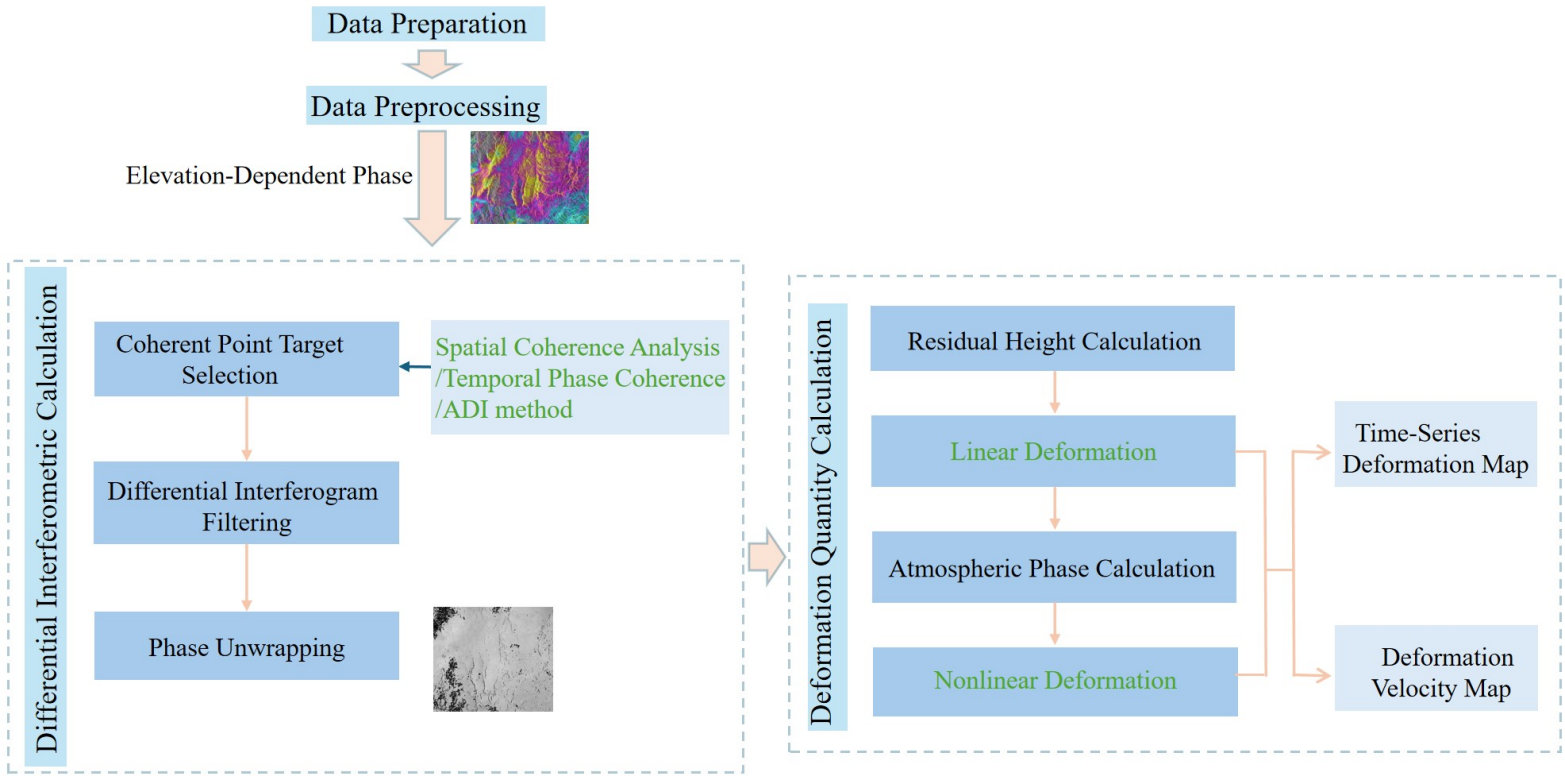
SBAS-InSAR Processing Workflow.

SBAS-InSAR is a Time-Series InSAR (TS-InSAR) technique based on a small baseline subset strategy. The overall workflow is illustrated in Figure 5: this method first requires the selection of coherent point targets, where relatively stable scatterers in landslide areas are identified to ensure monitoring accuracy using approaches such as phase stability-based selection, amplitude dispersion index, and spatial coherence analysis [96,97]. By constructing a network of interferometric pairs constrained by both spatial and temporal small baselines, SBAS-InSAR avoids severe decorrelation caused by long baselines, which constitutes its key advantage over D-InSAR and PS-InSAR. After phase unwrapping, residual height correction and atmospheric phase separation are further introduced to correct phase errors and separate linear and nonlinear deformation components, respectively, ultimately enabling the extraction of full time-series deformation characteristics of landslides.

From the perspectives of monitoring scenarios and deformation characteristics, SBAS-InSAR relies on multiple SAR images to construct time-series observations, making it more suitable for long-term slow landslide deformation monitoring than D-InSAR [98]. Meanwhile, its capability to handle nonlinear and high-gradient deformation is also superior to PS-InSAR. This advantage mainly stems from its multi-master-image small baseline subset strategy, which preserves a larger number of interferometric pairs and avoids decorrelation caused by single-master-image configurations [72]. In terms of accuracy and applicability conditions, SBAS-InSAR extracts signals from sparsely distributed scatterers with stable backscattering characteristics [69], and generally achieves higher accuracy than D-InSAR [99]. Compared with PS-InSAR, it shows better adaptability in complex terrain and vegetated areas. Relevant studies indicate that in suburban and low-coherence regions, the number of effective monitoring points obtained by SBAS-InSAR can reach 3.6 times that of PS-InSAR, with improved continuity of the deformation field, demonstrating stronger spatial coverage capability at regional scales [100]. In practical applications, SBAS-InSAR does not rely on manually selected permanent scatterers, requires less data volume, and offers higher computational efficiency. Therefore, it is more advantageous for natural landslide areas and wide-area landslide cluster monitoring [101].

However, SBAS-InSAR still exhibits notable scene-related and technical limitations in landslide monitoring, such as difficulties in selecting high-coherence points, high operational costs, complex processing procedures, and potential reductions in monitoring accuracy under conditions of strong nonlinear deformation or complex terrain. To address these issues, ref. [102] proposed TCP-InSAR, which effectively alleviates the difficulty of selecting high-coherence points and the problem of insufficient point density, thereby improving the applicability of surface deformation monitoring in complex regions. In addition, some studies have combined PS-InSAR and SBAS-InSAR to fully exploit the complementary advantages of the high coherence of PS points and the wide coverage of coherent targets in SBAS-InSAR. For example, Reference [103] successfully identified 36 potential landslide hazard sites using this integrated approach, with the monitored average displacement rates ranging from 57 mm/year (uplift) to −146 mm/year (subsidence). Reference [104] further integrated these two methods with ascending and descending SAR datasets to successfully extract the downslope deformation field of landslides, achieving a root mean square error (RMSE) of only 3.62 mm compared with in situ measurements, thereby demonstrating the superiority of multi-technique integration for detailed monitoring of critical landslides.

### 3.4. DS-InSAR Method

**Figure 6:**
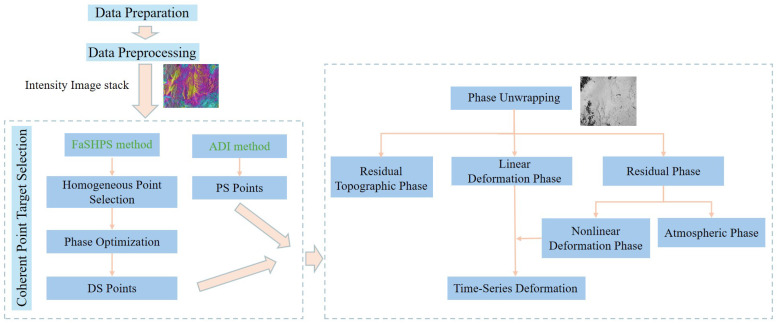
DS-InSAR Processing Workflow.

DS-InSAR is an MT-InSAR technique developed on the basis of PS-InSAR and represents a hybrid approach that integrates PS and Distributed Scatterers (DS) points. It significantly enhances the density of point targets in non-urban areas through the identification of statistically homogeneous pixels (SHPs) and phase optimization. The specific processing workflow is shown in Figure 6: the method first selects high-coherence PS candidate points using the ADT approach; simultaneously, the Fast Statistically Homogeneous Pixel Selection (FaSHPS) algorithm [105] is applied to identify clusters of homogeneous pixels within the study area, which are then converted into reliable DS through phase optimization. By fusing PS and DS points, the method expands the extraction of effective monitoring points across the landslide area. The extracted phases are subsequently optimized through filtering and noise removal to improve phase data quality, enabling accurate deformation retrieval in complex landslide regions.

From the perspectives of monitoring scenarios and deformation characteristics, the key advantage of DS-InSAR lies in overcoming the dependence of conventional PS-InSAR on permanent scatterers, significantly increasing the spatial density of monitoring points by jointly exploiting both PS and DS points [106]. At the same time, it avoids the spatial resolution loss caused by multi-looking processing in SBAS-InSAR and preserves the original resolution, making it more suitable for small-scale landslides. In long-term monitoring of water-rich landslides, the accuracy can be improved by more than 20% [107]. As a representative DS-InSAR technique, SqueeSAR was proposed by Ferretti et al. in 2011 [73]. A comparative study [108] evaluated SqueeSAR against PS-InSAR and SBAS-InSAR, showing that SqueeSAR increases the monitoring point density by approximately 3–5 times in vegetated areas and improves interferogram coherence by about 37%, fully demonstrating the applicability of DS-InSAR in landslide monitoring under low-coherence conditions. This indicates that under complex mountainous conditions with sparse monitoring points, DS-InSAR can provide more continuous deformation information than PS-InSAR and SBAS-InSAR. Another study [109] also confirmed that DS-InSAR achieves a higher measurement point density than both PS-InSAR and SBAS-InSAR, providing a more effective technical approach for surface deformation monitoring in both bare land and vegetated areas. However, since DS-InSAR requires simultaneous processing of both PS and DS targets, its computational cost is generally higher than that of PS-InSAR and SBAS-InSAR, and its efficiency remains limited in long time-series SAR data processing. To address this issue, reference [110] proposed an image compression-based DS-InSAR method, which reduces data volume while achieving improved monitoring performance, providing a new approach for long-term landslide monitoring in high-mountain and deep-valley regions.

To systematically compare the performance of the four classical InSAR techniques in landslide monitoring, we summarize their characteristics from five core dimensions, namely typical accuracy, computational efficiency, point density, vegetation adaptability, and application scope, as shown in Table 3.

### 3.5. Other Derived InSAR Methods

In addition to the classical InSAR methods discussed above, several derived InSAR techniques have emerged in recent years, each exhibiting specific advantages under different application scenarios. This section briefly introduces representative approaches, including GB-InSAR, QPS-InSAR, TCP-InSAR, and MAI, and analyzes their technical characteristics, applicable conditions, and typical applications in landslide monitoring. To provide a more intuitive comparison of their respective advantages, limitations, and monitoring capabilities, Table 4 summarizes and compares these derived methods (SqueeSAR has been introduced in Section 3.4).

GB-InSAR technology was first proposed by Tarchi et al. in 1999 and applied to dam structure monitoring, demonstrating its feasibility with sub-millimeter precision [111]. In 2003, it was first applied to landslide monitoring, using ground-based radar systems for close-range observation, offering high temporal resolution (on the order of minutes) and millimeter-level accuracy [112]. Its advantages include flexible deployment and the ability to obtain continuous spatial deformation with high precision. However, its application is still limited by loss of coherence in vegetated areas, phase errors induced by complex atmospheric disturbances, high cost, and restricted observation range, making it suitable primarily for monitoring single, well-defined landslides [113,114]. To this end, some studies [115] leveraged the high temporal resolution of GB-InSAR by combining it with spaceborne InSAR, forming a complementary monitoring strategy between regional screening and local detailed observation. Moreover, GB-InSAR also plays a crucial role in emergency early warning. For instance, reference [116] designed and implemented a landslide early-warning system, where GB-InSAR successfully captured the accelerated deformation phase of the San Leo landslide in Italy prior to its final collapse, triggering timely warnings and providing critical guidance for the evacuation of residents.

MAI was proposed by Bechor and Zebker in 2006. This technique extracts azimuthal surface deformation information, compensating for the limitation of traditional D-InSAR that can only measure line-of-sight (LOS) deformation, and can be used for three-dimensional deformation monitoring [117]. Its advantages include not requiring terrain phase removal or phase unwrapping, and the ability to suppress atmospheric phase delay. However, MAI is susceptible to decorrelation noise, residual phase in interferograms, and ionospheric disturbances, and targeted improvements have been proposed in [118,119,120]. Nevertheless, its applicability in low-coherence vegetated landslide areas remains limited, and the achievable accuracy is constrained; practical applications require consideration of the study area’s characteristics. For example, Ref. [121] proposed a coupled DInSAR-SBAS and MAI-SBAS framework, achieving high-precision time series inversion of two-dimensional deformation fields in steep slopes, overcoming the limitations of traditional InSAR, which only measures one-dimensional LOS deformation and is prone to geometric distortion in steep terrain.

QPS-InSAR was proposed in 2012 [122]. This technique employs a pixel-adaptive interferogram selection strategy to extract deformation information from partially coherent targets, i.e., scatterers between PS and DS, significantly increasing the measurement point density in non-urban areas [123]. Its advantage lies in mitigating spatiotemporal decorrelation to some extent, making it suitable for large-scale landslide screening and fault activity monitoring [124]. However, QPS-InSAR has relatively lower accuracy and is highly sensitive to spatial filtering strategies, and is therefore often used in conjunction with PS-InSAR [125]. Huang et al. applied QPS-InSAR to the Wanzhou area, successfully identifying multiple landslide deformations with a maximum rate of −21 mm/yr, validating its effectiveness in vegetated landslide regions [126]. Nevertheless, due to data volume limitations and sensitivity to spatial filtering, the method still requires improvements in capturing fine-scale deformation details and ensuring measurement stability.

TCP-InSAR was proposed in 2012. This method identifies Temporarily Coherent Points, i.e., pixels that remain coherent only within certain time windows, and adaptively selects temporal subsets, significantly extending InSAR applications in non-urban areas without requiring phase unwrapping [102]. The technique does not require phase unwrapping and is insensitive to orbital errors, making it suitable for slow landslide surveys with limited data. Zhang et al. successfully applied TCP-InSAR to extract long-term slow deformation features of landslides [127]. Its applicability in complex scenarios has also been demonstrated: in the Three Gorges Reservoir region, affected by reservoir water level fluctuations, TCP-InSAR detected deformation rates up to −60 mm/yr and revealed acceleration mechanisms during water level decline [128]. However, the method has limited accuracy in strongly nonlinear deformation areas and relies on a relatively dense stack of interferograms.

The above derivative methods each have their focus: QPS-InSAR and TCP-InSAR enhance monitoring capability over natural surfaces by optimizing scatterer identification strategies; GB-InSAR achieves local, high-resolution monitoring and early warning through platform innovation; MAI enables three-dimensional deformation retrieval by expanding the inversion dimension. In practical landslide monitoring, the choice of method—or a combination of methods—should be guided by landslide type, monitoring scale, and data availability.

### 3.6. Integration of AI and InSAR Methods

Although InSAR technology has been widely applied in landslide monitoring and has achieved considerable maturity, several inherent challenges remain, including difficulties in phase unwrapping, noise contamination, the handling of low-coherence areas, and the complexity of deformation time-series prediction [129]. In recent years, the rapid advancement of AI, particularly deep learning, has provided new technical opportunities to address these limitations. This section is organized around the key stages of InSAR data processing and landslide monitoring. It systematically reviews the targeted applications of various AI-based approaches—including CNNs, LSTMs, and Transformer-based models—in tasks such as phase unwrapping, noise suppression, intelligent landslide detection, and deformation trend prediction. Representative studies are further discussed to provide a critical assessment of their methodological advantages, limitations, and practical applicability.

(1) Phase Unwrapping and Noise Suppression

Phase unwrapping and noise suppression represent two fundamental challenges in InSAR data processing. Traditional approaches, such as the branch-cut method, the minimum cost flow algorithm, and filtering techniques including Lee filtering and Goldstein filtering, are prone to error accumulation, unwrapping failure, and fringe distortion when applied to low-coherence areas or regions experiencing large deformation gradients [64,130]. To address these limitations, researchers have increasingly introduced deep learning-based approaches to improve the robustness and accuracy of phase unwrapping and noise reduction.

According to the application paradigms of AI, existing phase unwrapping approaches can generally be categorized into two types: auxiliary-processing methods and end-to-end methods [131]. (i) Auxiliary-processing methods. These approaches employ AI to optimize specific steps within the conventional processing workflow. For example, the Discontinuity Estimation Network (DENet) model proposed in [132] predicts the probability of phase discontinuities using deep learning and incorporates this information into the cost-setting strategy of the minimum-cost flow unwrapping framework. Similarly, the Branch-Cut Network (BCNet) proposed in [133] reformulates the residue detection problem as a semantic segmentation task, enabling the intelligent deployment of branch cuts, after which the results are processed using the traditional branch-cut method. (ii) End-to-end methods. These approaches directly perform phase unwrapping or denoising tasks. PhaseNet 2.0, proposed in [134], reformulates phase unwrapping as a dense classification problem and employs a fully convolutional DenseNet to predict the wrapping counts, achieving accurate unwrapping even under strong noise conditions (−5 dB). The Unwrap-Net model proposed in [135] integrates airborne LiDAR data to assist phase unwrapping and achieves a structural similarity index (SSIM) of 0.91 and a RMSE of only 0.19 in simulation experiments. Even when the coherence decreases to 0.3, the model maintains strong performance (SSIM of 0.71 and RMSE of 0.37), significantly outperforming traditional methods such as the Goldstein branch-cut algorithm and SNAPHU (Statistical-Cost, Network-Flow Algorithm for Phase Unwrapping). To address scenarios with strong noise and complex fringe patterns, the Dilated Multi-Path Phase Unwrapping Network (DMP-PUNet) adopts a dilated multi-path architecture, improving accuracy by more than 50% on simulated datasets [136].

In terms of noise suppression, early PDNet leveraged phase self-similarity and applied a maximum likelihood approach to avoid the resolution loss and fringe distortion associated with traditional filtering methods, achieving near real-time denoising [137]. Generative approaches have also been introduced in this domain. Reference [138] proposed an unsupervised generative neural network that simultaneously performs InSAR phase filtering and coherence estimation through an encoder–decoder architecture, effectively suppressing noise while reducing over-smoothing artifacts near residual points. In addition, study [139] implemented phase filtering based on 3D CNNs, optimizing unwrapping compatibility during denoising, reducing residual points, and improving unwrapping reliability. Recently, hybrid architectures have emerged as a new direction. Hybrid Transformer-CNN Network (HTCNet), proposed in [140], employs a dual-branch parallel structure combining Transformer and CNN to extract global and local features, respectively, and adaptively fuse them, effectively removing speckle noise in SAR images while preserving textural details.

Traditional InSAR phase unwrapping and noise suppression methods are based on explicit physical models and mathematical optimization, offering advantages such as strong interpretability, no requirement for training datasets, and relatively low computational costs. These methods remain practical in scenarios with sufficient data availability and high coherence. However, in low-coherence regions, high-noise environments, and areas with complex deformation patterns, they are prone to error accumulation, unwrapping failures, and phase fringe distortion. In contrast, AI-based methods can automatically learn complex phase features from InSAR data and demonstrate improved robustness and processing accuracy in challenging scenarios. They can effectively reduce error accumulation and enhance overall processing performance. Nevertheless, AI methods still face several critical challenges. First, the quality and representativeness of training datasets remain insufficient, and the domain gap between simulated datasets and real SAR observations limits model generalization capability. Second, label uncertainty may introduce systematic biases, as reliable ground-truth phase unwrapping results are extremely difficult to obtain from real-world datasets. Third, the “black-box” nature of end-to-end models restricts their application in high-reliability scenarios, such as landslide early warning. Finally, the cross-regional transferability of AI models has not been sufficiently validated. For example, whether a phase unwrapping model trained in mountainous regions can be effectively applied to plain areas remains an open question requiring systematic investigation.

(2) Landslide Detection and Prediction

Landslide detection is a critical step for accurately delineating the locations and extents of landslides from the background. Traditional approaches rely on expert visual interpretation of InSAR deformation maps, which is inefficient, highly subjective, and limited in capturing nonlinear evolution patterns from large-scale time-series data. Artificial intelligence methods, by automatically learning spatiotemporal features of deformation, have significantly improved the accuracy of landslide detection and the foresight of deformation prediction [141].

Early studies primarily employed shallow models such as Random Forest (RF) [142] and Support Vector Machine [143], integrating InSAR deformation features with topographic, geological, and hydrological factors for landslide susceptibility assessment. Reference [144] combined SBAS-InSAR deformation rates with 13 factors including slope to achieve high-precision identification of slow-moving landslides in southern Italy using an RF model, confirming the critical role of deformation data in improving susceptibility mapping reliability. Reference [145] integrated MT-InSAR deformation rates with environmental factors and employed an eXtreme Gradient Boosting model to construct a landslide susceptibility mapping framework, achieving an Area Under the Receiver Operating Characteristic Curve (AUC) of 0.93–0.97, demonstrating that the combination of InSAR and machine learning can substantially enhance dynamic deformation capture and prediction accuracy. However, these approaches rely on manual feature engineering, insufficiently exploit spatiotemporal deformation patterns, and exhibit limited generalization due to the geographic specificity of the training areas. CNNs and their variants are the most widely used deep learning architectures for landslide detection. LeCun et al. [146] highlighted that CNNs, through local connectivity and weight-sharing mechanisms, can effectively extract spatial features from images. Reference [143] proposed U-Net, which employs an encoder–decoder structure with skip connections to achieve precise pixel-level segmentation while preserving spatial resolution. Networks such as U-Net and SegNet have been progressively applied to landslide detection tasks [147,148]. Reference [149] improved U-Net to accommodate InSAR data characteristics, developing a deformation-feature-based Deep Residual Shrinkage U-Net (DRs-UNet) deep semantic segmentation model for intelligent detection of active landslides in the Three Rivers Parallel Region of the Tibetan Plateau (Intersection over Union > 90%). Experiments indicate that DRs-UNet results are closer to actual landslide boundaries than U-Net and SegNet, outperforming them in terms of landslide morphology integrity and boundary continuity, visually demonstrating the capability of deep learning to automatically extract and classify InSAR deformation features. In addition, Reference [150] proposed an improved Faster Region-based CNN model that takes InSAR deformation rate maps as input to achieve automatic and accurate detection of slow-moving landslides in the Jinsha River basin (F1-score 93.6%), demonstrating good cross-regional generalization capability in the Tibetan Plateau. In recent years, Transformer architectures have brought new breakthroughs in landslide detection. Reference [151] introduced the Multiscale Swin Transformer InSAR Products Detection Network (MSIDNet) model, which employs a Swin Transformer to integrate multi-scale spatiotemporal attention mechanisms. By leveraging InSAR deformation rates, phase gradients, and gradient coherence indices, MSIDNet achieved precise detection of slow-moving landslides (accuracy 0.89), validating the ability of Transformer architectures to capture long-range dependencies in InSAR deformation features.

In terms of prediction, Reference [152] proposed a deep learning framework combining LSTM and Time-Gated Long Short-Term Memory (TGLSTM) for InSAR time-series change-point detection. By incorporating sampling intervals to address irregular sampling and using real-data simulations to overcome limited training data, the framework demonstrated effective detection of anomalous landslide displacements in practical monitoring projects. In addition, Reference [153] successfully integrated Gated Recurrent Unit (GRU) with MT-InSAR data for landslide prediction, verifying the superiority of GRU over LSTM. However, GRU is limited in modeling long sequences, while LSTM is computationally intensive and lacks interpretability. Building on this, Reference [154] proposed a coupling framework of MT-InSAR and deep learning, employing a CNN-Attention-Bidirectional Gated Recurrent Unit network to achieve high-precision prediction of landslide displacements in the Three Gorges Reservoir area (RMSE improved by 21–55% compared to conventional deep learning models). The attention mechanism also provides interpretability of the model’s decision process, extending the application of AI to dynamic evolution prediction of InSAR-derived deformation.

Compared with traditional InSAR-based landslide identification and prediction methods, which mainly rely on manual interpretation or handcrafted feature extraction, AI-based approaches can automatically learn complex spatiotemporal characteristics from InSAR deformation data, improving landslide detection efficiency, deformation prediction accuracy, and the characterization of nonlinear deformation evolution processes. However, AI models remain constrained by several limitations, including their dependence on high-quality training datasets, limited interpretability, and insufficient cross-regional generalization capability. In contrast, traditional methods still maintain practical value in data-scarce regions and complex scenarios requiring physical consistency constraints or expert knowledge. The accumulated experience of expert visual interpretation remains difficult to fully replace with current AI techniques. Therefore, AI should be regarded as a complementary approach to traditional methods rather than a complete replacement. Despite significant advances in existing AI-InSAR approaches, several systematic limitations remain. At the data level, InSAR deformation observations are susceptible to atmospheric delays, vegetation-induced decorrelation, and geometric distortions, resulting in unstable training data quality. At the methodological level, although end-to-end models achieve high accuracy, their “black-box” characteristics limit interpretability and their application in safety-critical scenarios. At the application level, existing studies are often conducted in individual regions or specific landslide types, while the cross-regional generalization capability and robustness under extreme climatic conditions remain insufficiently validated.

To address data bottlenecks such as the scarcity of labeled samples, foundation models pretrained on large-scale remote sensing imagery have provided new research perspectives for InSAR interpretation. Applied to tasks such as landslide identification, change detection, and deformation interpretation, these approaches have the potential to alleviate the scarcity of labeled InSAR data. For example, He et al. employed the Segment Anything Model to achieve zero-shot landslide detection from InSAR deformation rate maps, successfully identifying 54 landslides across multiple test areas, demonstrating the potential of foundation models for automated InSAR interpretation [155]. However, due to the unique scattering mechanisms and noise characteristics of SAR imagery, the adaptability of foundation models pretrained mainly on optical images in InSAR-related scenarios still requires further investigation.

### 3.7. Multi-Source Data Fusion Methods

A single data source is insufficient to fully characterize the complex evolution of landslides. By integrating InSAR with optical remote sensing, GNSS, meteorological data, and LiDAR, the comprehensiveness and reliability of landslide monitoring can be significantly improved. However, its practical effectiveness is still constrained by factors such as fusion strategy selection, spatiotemporal co-registration accuracy, and error propagation [156]. According to the fusion level, multi-source data fusion is generally classified into data-level fusion, feature-level fusion, and decision-level fusion [157]. In practical landslide monitoring, fusion strategies are generally designed according to specific monitoring objectives, and different tasks often require the combination of one or more fusion levels. Therefore, this section discusses multi-source data fusion from three major application objectives of landslide monitoring: landslide identification and boundary extraction, three-dimensional deformation monitoring, and landslide early warning and prediction, with a focus on the primary fusion strategies employed for each task.

(1) Fusion strategies for landslide identification and boundary extraction: primarily based on feature-level and decision-level fusion.

The core task of landslide identification is to determine the location, extent, and boundary characteristics of potential active landslides. Although InSAR provides high-precision deformation measurements, decorrelation frequently occurs in low-coherence areas, leading to loss of deformation information. Optical imagery offers high-resolution spatial localization and clear geomorphological texture information, enabling the identification of rear scarp cracks, landslide boundaries, and accumulation morphology, but it is less capable of capturing slow deformation processes [158]. For this task, existing studies mainly adopt feature-level and decision-level fusion strategies. The former integrates InSAR-derived deformation information with textural features from optical imagery to improve the accuracy of landslide identification and boundary extraction, while the latter combines detection results from multiple sources to further enhance the reliability of landslide mapping. For example, Reference [159] combined SqueeSAR with SPOT-6/7 optical imagery and derived a complete displacement field through pixel correlation analysis, increasing the completeness of landslide identification from 67% to 89%. Reference [160] integrated DS-InSAR with optical imagery in the Ahai reservoir area of the Jinsha River, identifying 15 landslides and achieving deformation classification. Reference [161] applied the integration of InSAR and optical remote sensing to loess landslide monitoring, further demonstrating the capability of InSAR for detecting early-stage creep deformation and the advantage of optical imagery in morphological interpretation. Related studies indicate that such strategies exhibit significant advantages in landslide identification under complex environments such as vegetated mountainous regions and loess areas [162]. The main challenges lie in geometric co-registration errors between heterogeneous datasets [163] and feature conflicts [164]. Future research still needs to focus on developing high-precision registration techniques and joint interpretation methods.

(2) Fusion strategies for three-dimensional deformation monitoring: primarily based on data-level fusion.

Landslide stability assessment requires not only the identification of landslide extent, but also the retrieval of actual movement direction, displacement rate, and internal deformation characteristics. However, InSAR typically provides only LOS displacement, making it difficult to fully reconstruct the three-dimensional motion process. GNSS provides high-precision point-based three-dimensional displacement observations, while LiDAR or DEM can offer geometric constraints of the slope surface and terrain information. For this task, data-level fusion is mainly applied to integrate multi-source observations, such as InSAR and GNSS, for three-dimensional displacement field reconstruction. Meanwhile, topographic geometric information provided by DEM data can be incorporated to constrain displacement inversion, thereby improving the reliability of three-dimensional deformation reconstruction. For example, Research [165] combines MT-InSAR and GNSS to perform LOS-to-3D displacement decomposition, revealing the tensile–compressive differentiation within the Jaggi Bagwan landslide. Research [166] integrates InSAR and GNSS to achieve joint surface–point monitoring, improving the accuracy of three-dimensional displacement estimation for urban slopes in Istanbul. Research [167] employs GNSS to constrain the PS-InSAR phase unwrapping network to eliminate residual phase errors, and derives slope and aspect from DEM to construct the geometric relationship of slope deformation, thereby reconstructing the three-dimensional displacement field. This approach was validated in the Jinpingzi landslide in the Wudongde reservoir area of the Jinsha River, achieving an accuracy of RMSE ±2.0–2.8 mm. These studies indicate that such strategies have been validated in scenarios including natural landslides in mountainous regions, urban slopes, and reservoir bank slopes. However, limitations such as sparse GNSS stations, differences in temporal sampling scales, and inconsistencies in reference frames still constrain large-scale application. To address these issues, Research [168] proposes a three-step data acquisition optimization algorithm to improve spatiotemporal matching efficiency, while Research [169] enhances GNSS station layout to support the unification of reference frames in low-coherence areas.

(3) Fusion strategies for early warning and prediction: primarily based on feature-level fusion, supplemented by decision-level fusion.

The key to landslide disaster prevention and control lies not only in detecting deformation but also in identifying its acceleration stage and predicting potential failure time. Meteorological factors such as precipitation and humidity are important triggers for landslides [170]. InSAR provides long-term deformation time series, while meteorological data reflect external driving conditions. For this task, feature-level fusion is mainly used to integrate deformation time series with external triggering factors, such as rainfall, temperature, and humidity, for coupled modeling of landslide evolution processes and spatiotemporal variations. Decision-level fusion can further improve the stability and robustness of early-warning systems by integrating prediction results from multiple models. For example, Research [171] integrates TS-InSAR deformation with hydrometeorological factors for modeling, revealing the environmental driving mechanisms of landslide deformation and providing a basis for early-warning analysis. Research [172] combines Complete Ensemble Empirical Mode Decomposition with Adaptive Noise (CEEMDAN) decomposition with meteorological lag information to predict InSAR deformation sequences, verifying the role of meteorological-deformation coupling in improving prediction accuracy. Research [173] integrates InSAR deformation with factors such as precipitation and temperature to construct a Two-Dimensional Temporal Convolutional Network model, and simulates eight extreme weather scenarios to identify high-risk states, validating the effectiveness of this strategy in risk assessment. This strategy shows good potential for landslide prediction and early warning in key risk points in monsoon regions with heavy rainfall. These studies indicate that meteorological-deformation fusion has evolved towards nonlinear modeling and scenario simulation. However, achieving a balance between prediction accuracy and physical interpretability remains a key challenge that requires further breakthroughs in this field.

In summary, research on multi-source data fusion is gradually shifting from early data overlay models to collaborative monitoring models aimed at specific task objectives. From the perspective of fusion hierarchies, different application tasks generally correspond to different dominant fusion strategies, with multiple fusion levels often being jointly employed. For regional landslide surveys and boundary identification, decision-level or feature-level fusion of InSAR and optical remote sensing should be prioritized. For high-precision three-dimensional displacement monitoring, data-level fusion of InSAR, GNSS, and DEM is the preferred approach. For trend prediction and short-term early warning, feature-level fusion of InSAR and meteorological factors offers significant advantages. These relationships indicate that data-level, feature-level, and decision-level fusion are not independent technical approaches, but rather complementary strategies that should be selected and combined according to specific monitoring requirements. Future efforts should focus on overcoming key challenges such as high-precision co-registration of heterogeneous data, cross-scale collaborative modeling, and physical mechanism constraints, to promote the development of multi-source fusion technologies towards higher precision, intelligence, and practicality.

## 4. Limitations and Challenges of InSAR Technology

Although InSAR technology offers advantages such as high precision, large coverage, and long-term continuous observation in landslide monitoring, its practical effectiveness is still constrained by factors such as imaging mechanisms, observation conditions, and complex surface environments. Particularly in mountainous areas, factors such as topographic variations, vegetation cover, water vapor changes, and complex motion patterns often affect monitoring accuracy and the reliability of results. Overall, the current limitations of InSAR in landslide monitoring primarily include geometric distortion, decorrelation noise, atmospheric delay errors, and difficulties in three-dimensional deformation monitoring. It should also be noted that the applicability of InSAR is closely related to the deformation rate of landslides: it is generally most effective for slow-moving landslides, whereas its applicability to rapidly evolving slope failures is inherently limited by its temporal resolution and coherence loss, an aspect further discussed in Section 4.4. These issues will be discussed in detail below.

### 4.1. Geometric Distortions

**Figure 7:**
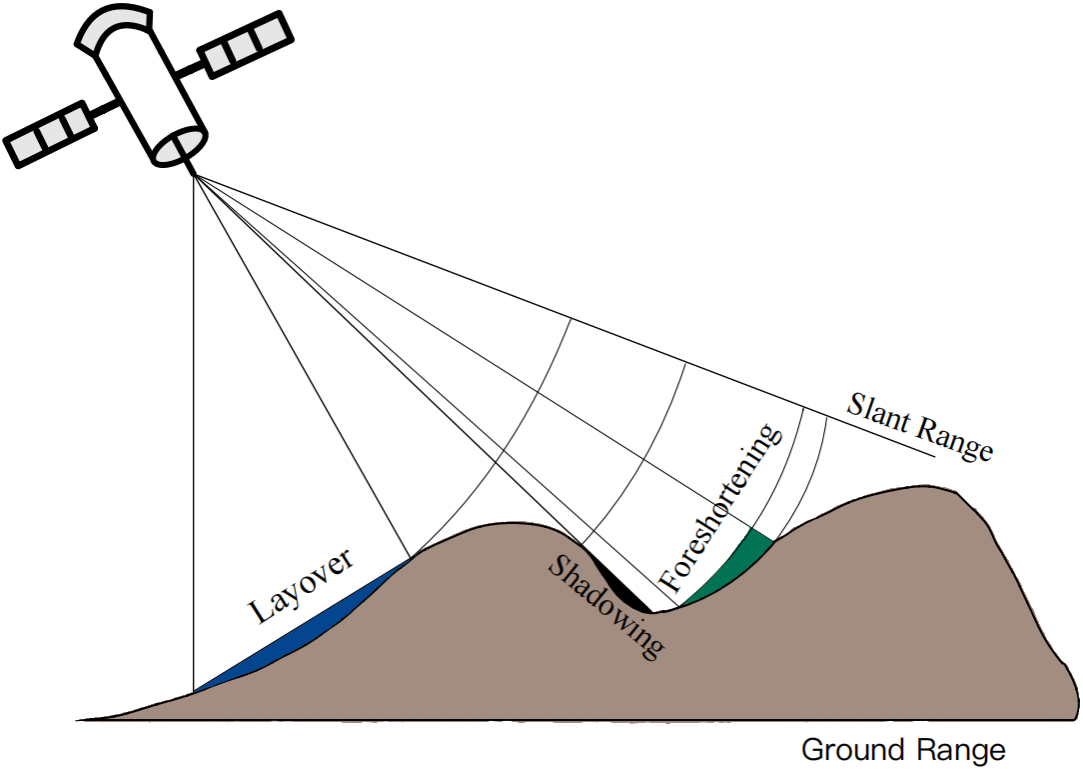
Schematic illustration of geometric distortions in SAR imaging.

SAR satellites use a side-looking imaging mode, and the interaction between the incident parameters and terrain features is a key factor in determining whether SAR can successfully acquire valid images [174]. The side-looking imaging mode is the main cause of geometric distortions, which include effects such as perspective compression, overlap, and shadowing (refer to Figure 7) [175]. Geometric distortions are particularly prominent in areas with steep terrain, such as high mountain gorges, deeply cut river valleys, and reservoir slopes, and are one of the key factors limiting the completeness of landslide identification and the continuity of long-term monitoring. These geometric distortions reduce the detection efficiency and application scope of SAR, creating observation blind spots that limit its effectiveness [176]. Studies have shown that geometric distortions significantly compress the effective observation range. For example, Research [177] simulated this effect across the entire UK and confirmed that 1.0–1.4% of the land area is rendered ineffective due to shadowing and overlap. In regions with more severe topographic variations (such as the Alps), the impact of geometric distortions is significantly amplified, severely restricting the completeness of landslide identification [178].

For landslide monitoring, if the landslide is located in shadow or layover areas, it may lead to missed detection of active landslides or incomplete boundary identification. Therefore, in practical applications, to reduce the impact of geometric distortions caused by layover and shadow on the accuracy of monitoring results, it is necessary to effectively determine the extent of these geometric distortion areas. This can typically be achieved through methods based on the relative geometric relationship between the sensor and the ground surface, such as the Layover-Shadow Map method [179], the Range-Index method [180], the Adjacent Gradient method [181], SAR signal-based statistical characteristic methods, Geographic Information System modeling methods [182], and spectral techniques [183].

After determining the extent of geometric distortion, it is necessary to further extract shadow and layover regions. For low-resolution SAR images, shadow and layover areas can be generated by integrating external DEMs with the geometric parameters of the SAR system. In contrast, for high-resolution SAR imagery, machine learning-based methods can be employed to accurately delineate layover and shadow regions [184]. Subsequently, TS-InSAR techniques are used to mask these layover and shadow areas, thereby reducing their interference in the phase unwrapping process. However, this approach inevitably leads to a reduction in the observable monitoring coverage [185]. To address this limitation, the complementary characteristics of ascending and descending orbit data in layover and shadow regions can be exploited to expand spatial coverage [186]. For example, Reference [185] achieved TanDEM-X ascending and descending DEM fusion, reducing the proportion of invalid pixels from 5–6% in single-orbit data to 2.14%, effectively mitigating the impact of geometric distortions and demonstrating the feasibility of extending coverage through ascending–descending data integration.

Nevertheless, geometric distortion has not been fundamentally resolved. On the one hand, ascending and descending orbit data cannot be synchronously acquired in all regions, leading to limited data availability; on the other hand, in extremely steep slopes or deeply incised valleys, permanent blind areas may still exist even with multi-orbit observations, preventing effective monitoring of critical parts of landslide bodies. In addition, regarding the two types of geometric distortions—foreshortening and resolution enhancement—the data quality in the resolution enhancement areas is generally higher, whereas the spatial resolution in foreshortening areas is relatively lower. However, there is still a lack of in-depth research on how to accurately evaluate the signal quality and usability of both foreshortening and resolution enhancement regions [176].

### 4.2. Decorrelation Noise

Decorrelation noise arises when radar signals lose coherence between images acquired at different times due to multiple factors, such as temporal baselines, dense vegetation cover, and large surface deformation gradients, resulting in noise signals. This type of noise interferes with the accurate extraction of true surface deformation signals, thereby degrading the accuracy of InSAR measurements. In landslide monitoring, this issue mainly occurs in two typical scenarios: temporal decorrelation caused by vegetation coverage and deformation-gradient decorrelation induced by rapid ground motion. Its generation mechanisms are closely related to surface land-cover types and deformation characteristics, and the decorrelation mechanisms as well as corresponding mitigation strategies vary significantly across different scenarios.

For landslide areas with dense vegetation cover, the influence of vegetation on coherence is closely related to parameters such as radar wavelength, spatial resolution of SAR imagery, and temporal–spatial baselines. Specifically, the L-band (wavelength 23.6 cm) exhibits stronger vegetation penetration capability and better resistance to temporal decorrelation than the C-band (5.6 cm), enabling the acquisition of richer information beneath the vegetation canopy. This improves the accuracy of coherence estimation and makes L-band more suitable for repeat-pass surface observation studies [187]. However, in densely vegetated regions, even high-resolution SAR data may still lead to failure of D-InSAR due to decorrelation and large deformation gradients. In such cases, sub-pixel offset tracking can serve as an effective alternative monitoring technique [84]. Alternatively, enhanced TS-InSAR methods such as SBAS-InSAR and SqueeSAR [73] can be employed. These approaches jointly exploit distributed scatterers to increase the number of coherent observations and enhance the signal-to-noise ratio [188], thereby recovering deformation information over a wider area. Some studies have also explored phase optimization using single-polarization SAR; however, the results have generally been unsatisfactory. With the development of polarimetric SAR, new approaches have been proposed and have achieved improved performance. For instance, the Sequential Estimation and Total Power-Enhanced Expectation–Maximization Inversion algorithm proposed in [189] integrates a sequential estimator [190] with a total-power polarimetric stacking strategy, and employs a maximum likelihood estimator based on eigen-decomposition for solution. Compared with conventional EMI methods, it increases the number of coherent points by more than 50%, providing valuable insights for real-time monitoring and decision-making.

For regions characterized by large-gradient deformation induced by landslides, earthquakes, and other processes (deformation rate > 5 m/yr), although L-band SAR is capable of capturing larger deformation magnitudes, L-band interferometric observations alone are still insufficient to meet the requirements of large-gradient deformation measurement. To overcome phase decorrelation caused by strong deformation gradients in conventional interferometry, SAR POT and range split-spectrum interferometry (RSSI) techniques can be jointly employed for measuring large deformation gradients [191]. The integrated use of multiple techniques and methodologies can effectively mitigate the impact of decorrelation noise, thereby improving the accuracy and reliability of InSAR-based monitoring [184].

Although the aforementioned methods can alleviate the impact of decorrelation noise to some extent, decorrelation remains fundamentally unresolved in extremely vegetated areas or regions with large-gradient deformation. Specifically, in vegetation-covered areas, even L-band SAR may become ineffective under extreme vegetation conditions such as tropical rainforests. In addition, the selection of optimal acquisition temporal pairs lacks systematic quantitative studies. The acquisition of polarimetric SAR data is also limited, while the associated algorithms are computationally complex. In regions with large-gradient deformation, the accuracy of POT is significantly lower than that of phase-based methods, and its spatiotemporal fusion with InSAR has not yet been fully developed. Moreover, the applicability of RSSI remains limited. Overall, existing methods are mostly optimized for specific scenarios, and a unified framework applicable to diverse landslide environments is still lacking. Future research should further explore multi-band SAR collaborative observations, adaptive coherence restoration algorithms, and an evaluation framework for method applicability under extreme conditions, in order to enhance the monitoring capability of InSAR in complex surface environments.

### 4.3. Atmospheric Delay Errors

Atmospheric delay errors refer to the variations in electromagnetic wave propagation speed caused by the spatial and temporal heterogeneity of atmospheric physical properties (such as temperature, humidity, and pressure) as the waves pass through the atmosphere, resulting in ionospheric and tropospheric delays. Ionospheric delay is primarily induced by variations in free electron density in the upper atmosphere, whereas tropospheric delay is associated with factors such as water vapor, temperature, and atmospheric pressure [192]. Reference [70] indicates that spatial and temporal variations in relative humidity of 20% can lead to deformation product errors of approximately 10 cm. In landslide monitoring, this issue is particularly prominent in humid climate regions and monsoon areas, where strong spatiotemporal variability of tropospheric water vapor represents a major source of error in high-precision InSAR observations.

To address this issue, various approaches have been proposed, including external data-assisted correction, TS-InSAR techniques, spatiotemporal filtering methods, and analyses of the spatiotemporal characteristics of atmospheric phase delays. For instance, external datasets can be used to estimate and suppress atmospheric noise in InSAR processing [193]. Reference [194] developed a water vapor correction model for InSAR atmospheric correction by integrating GPS, Moderate Resolution Imaging Spectroradiometer (MODIS), and InSAR data, significantly reducing the impact of atmospheric water vapor on InSAR measurements and improving the accuracy of surface deformation monitoring. Reference [195] further combined GPS and MODIS water vapor products and applied zonal calibration and kriging interpolation, achieving a reduction in atmospheric phase Root Mean Square by 32.74–38.82% in experiments. Alternatively, spatiotemporal filtering methods can be adopted. Reference [196] pointed out that when applying TS-InSAR filtering techniques, seasonal effects of atmospheric delay should be considered, and emphasized that appropriate selection of filtering parameters is crucial for mitigating atmospheric delay effects. In addition, TS-InSAR methods and analyses of atmospheric phase spatiotemporal characteristics have also been widely used. Reference [197] employed ERA-5 data and established a periodic zenith total delay negative exponential function model to characterize atmospheric spatiotemporal variations, which reduces temporal oscillation errors caused by tropospheric delay and improves the accuracy of TS-InSAR in retrieving surface deformation. The effectiveness of this model was validated through multiple evaluation metrics. Beyond these approaches, atmospheric phase can also be estimated using independent component analysis (ICA) [198]. The advantage of this method is that it does not require explicit functional relationships between observations and unknown parameters, nor does it rely on external data, yet it can achieve effective signal separation [199]. It should be noted that ICA is suitable for regions lacking external meteorological data; however, its performance is limited when deformation signals and atmospheric signals overlap in the spatiotemporal frequency domain.

Although various correction strategies have been developed, many challenges still remain. On the one hand, meteorological stations in mountainous regions are sparsely distributed, and the spatial resolution of external datasets is limited. On the other hand, when true deformation signals overlap with atmospheric noise in the spatiotemporal frequency domain, the effectiveness of filtering and blind source separation methods decreases significantly. Future research should focus on the assimilation of high-resolution meteorological reanalysis data, constraints based on tropospheric propagation mechanisms in complex mountainous terrains, and atmospheric correction approaches that integrate physical models with machine learning, in order to improve the applicability and robustness of InSAR in complex topographic environments.

### 4.4. Challenges in Three-Dimensional Deformation Monitoring

Surface deformation associated with landslides is highly complex and involves multiple deformation components, including both horizontal and vertical movements. In alpine canyon regions, deeply incised valleys, and large accumulation landslides, accurate characterization of three-dimensional motion is crucial for stability assessment. However, conventional InSAR techniques acquire data from a single viewing geometry and mainly measure displacement along the satellite LOS, making it difficult to accurately separate and quantify deformation components in different directions. Retrieving true three-dimensional deformation generally requires at least three groups of observations with significantly different imaging geometries [18]. In practical applications, however, the requirements for temporal synchronization and geometric diversity are often difficult to satisfy due to limitations imposed by satellite revisit cycles, orbital parameters, and imaging modes [200]. In addition, geometric distortions in complex terrain, such as shadowing and foreshortening, further reduce the availability of usable observations [201], leading to insufficient observation equations or unfavorable geometric configurations for three-dimensional inversion, thereby increasing solution uncertainty. According to the completeness of observation data, this section discusses the main existing approaches for three-dimensional deformation monitoring from two perspectives: data-driven methods and prior-constrained methods.

In regions where multi-orbit SAR data are relatively abundant and ground-based monitoring conditions are available, data-driven approaches are commonly adopted to improve three-dimensional deformation retrieval capability. For example, multi-orbit D-InSAR joint inversion can integrate ascending, descending, and multi-band SAR data to enhance observation geometry. In addition, InSAR can be integrated with GPS measurements. Reference [202], for instance, combined InSAR and GPS data to investigate the Crescent Lake landslide, retrieving thickness variations, sliding surface morphology, and the relationship between landslide motion and rainfall, thereby providing more comprehensive three-dimensional deformation information. Furthermore, POT and MAI techniques [203] can be incorporated to capture azimuthal deformation and increase observational dimensions. Reference [204] employed high-resolution TerraSAR-X data and an improved point target (PT) offset tracking method, using artificial corner reflectors to derive the spatiotemporal deformation patterns of a landslide, thereby demonstrating the feasibility of this approach. However, these methods each have inherent limitations: multi-orbit D-InSAR exhibits low accuracy in the north–south direction; the integration of D-InSAR and GPS relies on ground observation stations, which are costly and sparsely distributed; and MAI is highly sensitive to decorrelation noise, with relatively few successful application cases reported.

In remote mountainous regions, where SAR observations are sparse or the observation geometry is constrained, prior information on landslide behavior is commonly introduced as a constraint to reduce the dependence of inversion on diverse observation geometries. It should be emphasized that such geological priors fundamentally represent the long-term deformation mechanisms of geomaterials under gravity-driven loading, and their mathematical formulation can generally be described within a rheological framework. Specifically, geological constraints [205], such as landslide boundaries and rock stratum orientations derived from geological surveys, can be used to constrain the spatial extent and direction of deformation. Assumed sliding directions [206], inferred from topographic characteristics, can decompose three-dimensional deformation into slope-parallel and slope-normal components, thereby reducing the number of unknown parameters [207]. In addition, kinematic models [208], including rigid-body translation, rotational motion, or physics-based landslide motion models, can establish mathematical relationships among deformation components and supplement the observation equations. The Slope-Parallel Flow (SPF) model is a representative geological prior constraint model whose fundamental concept originates from the laminar flow approximation used in glacier and rock glacier dynamics [209]. This model simplifies a landslide mass as a thin layer flowing parallel to the slope surface. By establishing geometric constraints between the vertical and horizontal velocity components, it enables three-dimensional motion estimation using only ascending and descending InSAR observations [210,211]. Building upon this framework, Reference [212] further introduced a strain model to develop the SPF-constrained strain model method, integrating multi-source InSAR data for complete three-dimensional deformation inversion and demonstrating the effectiveness of combined prior constraints in improving inversion stability. However, the effectiveness of this approach depends critically on the validity of the underlying prior assumptions. As highlighted by the classical rheological framework, models are inherently approximations of real physical systems [213]. If the imposed constraints are inconsistent with the actual kinematic behavior of a landslide—for example, applying the SPF laminar-flow assumption to landslides dominated by rotational sliding, toppling, or debris-flow motion—the inversion results may exhibit systematic biases. Furthermore, the transferability of geological priors across different landslide types has not yet been quantitatively evaluated, highlighting the need for rigorous validation using independent field observations. In addition, the high cost of geological investigations limits the large-scale application of this approach.

Although the aforementioned methods have advanced three-dimensional deformation monitoring to some extent, several common challenges remain in practical engineering applications, including the difficulty of spatiotemporal registration for multi-source datasets, the limited accuracy of north–south deformation inversion, and the lack of a comprehensive validation framework for prior constraints. These issues continue to hinder the operational implementation of three-dimensional deformation monitoring. Regarding data registration, the use of standardized geographic coordinates expressed as the tuple [decimal lat, decimal long], denoted as [dLL], can alleviate geographic reference inconsistencies induced by the conventional degrees–minutes–seconds format, thereby facilitating precise site identification and providing a nonlocal reference framework for Lagrangian data modeling [214,215]. Therefore, appropriate three-dimensional deformation-monitoring methods should be selected according to landslide type, monitoring objectives, and available datasets. Future research should place greater emphasis on multi-platform collaborative observations, adaptive constraint optimization, and uncertainty quantification for three-dimensional deformation reconstruction, thereby improving the accuracy and reliability of landslide deformation monitoring in complex terrain.

However, the above discussion primarily applies to slow-moving landslides. For rapidly evolving landslides characterized by abrupt acceleration and high movement velocities, the effectiveness of InSAR is constrained by its limited temporal resolution and rapid decorrelation [176]. Moreover, temporal inconsistencies among multi-orbit or multi-platform observations may further exacerbate phase unwrapping errors and noise, thereby reducing the accuracy and reliability of three-dimensional deformation reconstruction [216]. In such scenarios, complementary monitoring techniques with higher temporal resolution, such as real-time GNSS positioning, terrestrial laser scanning, and unmanned aerial vehicle photogrammetry, may be more suitable for capturing the pre-failure stage and the failure process of landslides.

## 5. Conclusions and Future Directions

InSAR technology, with its wide-area coverage, high-precision measurement capability, and all-weather, day-and-night observation advantages, has become an important tool for landslide monitoring and disaster assessment. This paper systematically reviews the research progress of InSAR techniques in landslide monitoring and summarizes the development and applications of multi-temporal InSAR methods, including D-InSAR, PS-InSAR, SBAS-InSAR, and DS-InSAR. Existing studies demonstrate that multi-temporal InSAR techniques can achieve millimeter-level surface deformation monitoring and play an important role in regional-scale landslide identification, deformation evolution analysis, and disaster risk assessment. At the same time, the integrated use of InSAR with multi-source information, such as optical remote sensing, GNSS, LiDAR, and meteorological data, is promoting the transition of landslide monitoring from single-deformation observation toward multidimensional sensing and comprehensive interpretation. However, challenges such as geometric distortion, decorrelation, atmospheric delay, and insufficient three-dimensional deformation inversion capability still affect monitoring accuracy. Moreover, most current studies remain focused on post-disaster identification or retrospective analysis, while operational applications aimed at continuous monitoring and proactive early warning still require further development.

Future research on InSAR-based landslide monitoring may focus on the following aspects:

(1) Establishing automated and near-real-time landslide-monitoring systems: By leveraging open-access SAR datasets (e.g., Sentinel-1) and continuously updated global remote sensing resources, combined with cloud computing platforms and high-performance computing architectures, it is possible to achieve automated workflows from data acquisition and interferometric processing to deformation analysis. Such systems could support regional- to global-scale identification of landslide anomalies and near-real-time early warning in key mountainous areas, thereby improving rapid-response capabilities on timescales from minutes to hours. (2) Strengthening the synergistic integration of InSAR and optical remote sensing data: InSAR is effective for acquiring high-precision deformation information, whereas optical imagery has advantages in landslide boundary delineation, crack tracking, surface-cover change detection, and rapid post-disaster investigation. Future studies should promote the integration of these datasets at the data, feature, and decision levels to achieve comprehensive analyses linking “deformation monitoring–boundary identification–activity tracking”, thereby improving landslide detection and interpretation under complex conditions. (3) Developing AI-driven continuous tracking and prediction models: Building upon existing studies on automated identification, future research should further integrate InSAR time series with rainfall, topographic, geological, and historical hazard data to construct machine learning and deep learning models, such as Transformer-based and graph neural network spatiotemporal frameworks. These approaches could enable accelerated anomaly detection, deformation trend prediction, and dynamic risk-level assessment for landslides. (4) Enhancing the coupling between InSAR observations and physical models: Current research still places insufficient emphasis on landslide evolution mechanisms. Future work should integrate deformation time series derived from InSAR with hydrological process models (e.g., rainfall infiltration), groundwater response models, and geotechnical mechanical models (e.g., limit equilibrium analysis or finite element analysis) in order to improve understanding and prediction of landslide initiation, acceleration, and failure processes: (5) Improving monitoring robustness and model interpretability under complex environmental conditions. For alpine canyon regions, densely vegetated areas, and regions frequently affected by clouds and rainfall, further improvements are needed in decorrelation suppression, atmospheric delay correction, and three-dimensional deformation inversion methods. At the same time, enhancing the interpretability and cross-regional generalization capability of AI models will facilitate the transition of research outcomes toward operational applications: (6) Promoting standardized georeferencing and Lagrangian data modeling frameworks. Future studies should adopt [dLL] as a unified spatial reference to reduce errors associated with coordinate format conversion and improve the comparability of multi-source observations through a unified spatial indexing framework. This standardized representation also facilitates the integration of external validation datasets and can be combined with Lagrangian tracking frameworks to support continuous deformation analysis.

Overall, future research should promote the transition of InSAR applications from post-disaster identification and static monitoring toward near-real-time sensing, continuous tracking, mechanism analysis, and risk prediction. Through automated processing, multi-source data integration, and the combination of data-driven approaches with physical mechanism models, a more comprehensive landslide-monitoring and early-warning framework can be established to provide reliable technical support for disaster risk management.

## Figures and Tables

**Table 1:** Key Parameters and Applications of Commonly Used SAR Satellites (Part 1).

Satellite	Launch	Revisit (Day)	Mode & Swath (km)	Resolution (m)	Band (cm)	Cost & Use
TerraSAR-X	2007.06	11	HS: 10 SL: 10 SM: 30 SC: 100	1 × (1.5–3.5) 2 × (1.5–3.5) 3 × (3–6) 26 × 16	X (3.11)	High cost; detailed monitoring of individual landslides and steep rock slopes
COSMO-SkyMed	CSK-1: 2007.06 CSK-2: 2007.12 CSK-3: 2008.10 CSK-4: 2010.11	24	SL: 10 SM: 30–40 SC: 100–200	1 × 1 13–15 30–100	X (3.12)	Commercial data; small-scale landslide monitoring and boundary mapping
RADARSAT-2	2007.12	24	SL: 20 SM: 20–150 SC: 300–500	0.8 × (2.1–3.3) (3–25.6) × (2.5–42.8) (46–113) × (43–183)	C (5.63)	Commercial data; moderate cost; small-scale landslide monitoring and regional screening
TanDEM-X	2010.06	11	HS: 10 SL: 10 SM: 30 SC: 100	1 × (1.5–3.5) 2 × (1.5–3.5) 3 × (3–6) 26 × 16	X (3.11)	Commercial data; detailed landslide monitoring and DEM-assisted deformation analysis
Sentinel-1A	2014.04	12	SM: 80 IW: 250 EW: 400 Wave: 20	5 × 5 5 × 20 20 × 40 5 × 5	C (5.63)	Free open data; regional landslide detection and rainfall-triggered monitoring
ALOS-2	2014.05	14	SL: 25 SM: 50/70 SC: 350/490	1 × 3 3, 6, 10 100/60	L (23.5)	Moderate cost; landslide monitoring in vegetated areas and long-term deformation analysis
ICEYE constellation	2018.01	1–3	SF: 5 × 5 DP: 5 × 5 SM: 30 × 50 SW: 200 × 300	0.5 × (0.39–0.73) 0.25 × (0.23–0.36) 3 × 3 27 × 27	X	On-demand commercial data; high-frequency monitoring and emergency response
PAZ	2018.02	11	SM: 30/15 SC: 100 WS: 196–273 SL: 10 HS: 5–10 ST: 4.6–9	3.05 × (2.99–3.52) (18.5 × 16.79)–18.19 38.27 × 35 (1.46 × 1.55)–3.43 (1.05 × 1.0)–1.76 (0.38–0.7) × (0.96–1.78)	X (3.11)	Commercial data; coordinatedwith TerraSAR-X/TanDEM-X fordetailed monitoring
Capella constellation	2018.12	1–3	SLU: 5 × 5 SL: 5 × 5 SLW: 10 × 20 SM: (5–8) × (20–100) PSM: 20 × 20 /50/100	0.25 × (0.25/0.3) 0.5 × (0.25/0.3) 1.0 × 0.5 1.2 × 0.75 1.2 × 0.75	X	Commercial high-resolution data; pre-failure monitoring and rapid assessment

**Table 2:** Key Parameters and Applications of Commonly Used SAR Satellites (Part 2).

Satellite	Launch	Revisit (Day)	Mode & Swath (km)	Resolution (m)	Band (cm)	Cost & Use
GF-3	2016.08	29	12 modes: 10–650	1–500	C (5.63)	Public data; mountainous landslide monitoring; regional risk assessment
GF-3B	2021.11	5 h (three-satellite modeconstellation)	Additional UFS mode	1–500 0.5 × 0.5	C + L (5.63, 23.5)	Moderate cost; multi-baseline interferometry for detailed deformation monitoring
GF-3C	2022.04	5 h (constellation)	Same as GF-3	1–500	C (5.63)	Public data; mountainous landslide monitoring; regional risk assessment
SAOCOM-1B	2020.08	16	SM: 20–40 TN: 100–150 TW: 220–350	10 × 10 (30–50) × (30–50) (50–100) × (50–100)	L (23.5)	Commercial data; strong vegetation penetration; global landslide monitoring
LT-1A/B	1A: 2022.01 1B: 2022.02	4 (dual-satellite)	SM1: 50 SM2: 100 SM3: 50 SM4: 30 SM5: 150–250 SC: 400	3 12 3 6 20–30 30	L (23.5)	Strong vegetation penetration; landslide monitoring in vegetated areas and long-term observation
Chaohu-1	2022.02	12	SL: 7 × 7 SM: 25 SC: 100/170	1 3 12/20	C	Commercial data; landslide/geohazard emergency monitoring; ocean monitoring; agricultural remote sensing
Hongtu-1	2023.03	15	SL: 5 × 5 SSL: 10 × 20 SM: 20–30 TS: 50	0.5 × (0.45–1.03) 1 × (0.83–1.03) 3 × (<3) 5 × (<5)	X (3.11)	Commercial data; landslide monitoring; ground subsidence; 3D topographic mapping; emergency mapping
LT-4-01	2023.08	<4 h (regional)	GEO-SAR (>1000)	20–100	L (23.5)	Moderate cost; near-real-timemonitoring and rapid disaster assessment
NISAR	2025.07	12	SWP: 242	7 × (2–8)	L + S (24, 9.4)	Free open data; global landslide, glacier and vegetation deformation monitoring

Note 1: SM = StripMap, SC = ScanSAR, WS = Wide ScanSAR, SL = Spotlight, HS = HR Spotlight, ST = Staring Spotlight, TN = TopSAR Narrow, TW = TopSAR Wide, SLU = Spotlight Ultra, SLW = Spotlight Wide, PSM = Parallel Stripmap, SF = Spot Fine, DP = Dwell Precise, SW = Scan Wide, UFS = Ultra-Fine StripMap, GEO-SAR = Geosynchronous SAR, IW = Interferometric Wide, EW = Extra Wide, SSL = Sliding-Spotlight, TS = TOPSAR, SWP = SweepSAR. Note 2: Only active and commonly used satellites are included in this table.

**Table 3:** Comparison of Representative InSAR Techniques for Landslide Monitoring.

Technique	Accuracy	Computational Efficiency	Point Density	Vegetation Adaptability	Monitoring Scope
D-InSAR	cm level	High	Areal coverage (affected by decorrelation)	Poor	Short-term rapid deformation triggered by earthquakes or rainfall
PS-InSAR	mm to sub-mm	Low	High in urban areas (100–300 PS/km^2^); low in vegetated areas (41 PS/km^2^, Ancona landslide)	Fair	Urban and hard-target areas; slow-moving creep-type landslides
SBAS-InSAR	mm level	Moderate	Moderate (up to 3.6 times that of PS-InSAR in low-coherence areas)	Good	Natural landslide areas; regional-scale monitoring
DS-InSAR	mm level	Low	High (approximately 5 times PS-InSAR and 2.8 times SBAS-InSAR in Masuleh landslide)	Very good	Vegetated mountainous areas; small-scale landslide monitoring

**Table 4:** Comparison of Extended InSAR Techniques for Landslide Monitoring.

Technique	Typical Accuracy	Computational Efficiency	Temporal/Spatial Resolution (TR/SR)	Advantages	Limitations	Monitoring Scope
GB-InSAR	sub-mm	High (real-time)	TR: minute-level (continuous observation); SR: meter–sub-meter	Low temporal decorrelation, continuous real-time observation, low sensitivity to external influences	No mature atmospheric correction model, severe decorrelation in vegetated areas	Individual landslides, deep-seated surface landslides
MAI	cm level (can reach mm level when combined with other techniques)	Moderate	TR: same as DInSAR/SBAS; SR: same as original SAR images	No need for terrain phase removal, suppresses atmospheric phase delay	Sensitive to decorrelation noise, ionospheric disturbances, residual phase in interferograms	3D deformation measurement
SqueeSAR	mm level	Low	TR: depends on satellite revisit cycle; SR: same as original SAR images	Extracts deformation in sparsely vegetated areas, high data utilization, not strictly dependent on short baseline combination	Not suitable for large-area monitoring, heterogeneous pixel interference	Landslide mapping, mountainous landslides
QPS-InSAR	mm level	Moderate	TR: depends on satellite revisit cycle; SR: same as original SAR images	Uses spatial filtering to alleviate insufficient PS points in non-urban areas	Limited accuracy, sensitive to spatial filtering strategies	Large-scale preliminary landslide screening, fault monitoring; suitable for low-coherence vegetated areas
TCP-InSAR	mm level	High	TR: depends on satellite revisit cycle; SR: same as original SAR images	No phase unwrapping required, optimized vertical baseline, high monitoring accuracy	No matching high-resolution DEM, assumes linear deformation	Slow-moving landslide investigation

## Data Availability

No new data were created or analyzed in this study.

## Abbreviations

The following abbreviations are used in this manuscript:

InSAR	Interferometric Synthetic Aperture Radar
D-InSAR	Differential Interferometric Synthetic Aperture Radar
PS-InSAR	Persistent Scatterer Interferometric Synthetic Aperture Radar
SBAS-InSAR	Small Baseline Subset Interferometric Synthetic Aperture Radar
DS-InSAR	Distributed Scatterer Interferometric Synthetic Aperture Radar
QPS	Quasi-Persistent Scatterer
TCP-InSAR	Temporarily Coherent Point Interferometric Synthetic Aperture Radar
MAI	Multiple Aperture Interferometry
AI	Artificial Intelligence
POT	Pixel Offset Tracking
SAR	Synthetic Aperture Radar
PALSAR	Phased Array type L-band Synthetic Aperture Radar
RCM	Radarsat Constellation Mission
GPS	Global Positioning System
DEM	Digital Elevation Model
NL-InSAR	Non-Local Interferometric Synthetic Aperture Radar
MT-InSAR	Multi-Temporal Interferometric Synthetic Aperture Radar
GB-InSAR	Ground-Based Interferometric Synthetic Aperture Radar
StaMPS	Stanford Method for Persistent Scatterers
ADSI-CLC	Adaptive Distributed Scatterer InSAR Combined with Land Cover
GNSS	Global Navigation Satellite System
CNNs	Convolutional Neural Networks
LSTMs	Long Short-Term Memory Networks
PS	Permanent Scatterers
ADT	Amplitude Dispersion Threshold
CC	Coherence Coefficient
LAMBDA	Least-Squares AMBiguity Decorrelation Adjustment
RMSE	Root Mean Square Error
DS	Distributed Scatterers
SHPs	Statistically Homogeneous Pixels
FaSHPS	Fast Statistically Homogeneous Pixel Selection
QPS-InSAR	Quasi-Permanent Scatterer Interferometric Synthetic Aperture Radar
LOS	Line-of-Sight
DENet	Discontinuity Estimation Network
BCNet	Branch-Cut Network
LiDAR	Light Detection and Ranging
SSIM	Structural Similarity Index
DMP-PUNet	Dilated Multi-Path Phase Unwrapping Network
RF	Random Forest
HTCNet	Hybrid Transformer-CNN Network
AUC	Area Under the Receiver Operating Characteristic Curve
DRs-UNet	Deep Residual Shrinkage U-Net
TGLSTM	Time-Gated Long Short-Term Memory
MSIDNet	Multiscale Swin Transformer InSAR Products Detection Network
GRU	Gated Recurrent Unit
CNN-Attention-BiGRU	CNN-Attention-Bidirectional Gated Recurrent Unit
SAM	Segment Anything Model
CEEMDAN	Complete Ensemble Empirical Mode Decomposition with Adaptive Noise
TS-InSAR	Time-Series Interferometric Synthetic Aperture Radar
RSSI	Range Split-Spectrum Interferometry
MODIS	Moderate Resolution Imaging Spectroradiometer
ICA	Independent Component Analysis
SPF	Surface-Parallel Flow
SLC	Single-Look Complex
